# Complete mitochondrial genome assembly of *Zizania latifolia* and comparative genome analysis

**DOI:** 10.3389/fpls.2024.1381089

**Published:** 2024-08-09

**Authors:** Xianyang Luo, Cuicui Gu, Sijia Gao, Man Li, Haixiang Zhang, Shidong Zhu

**Affiliations:** College of Horticulture, Anhui Agricultural University, Hefei, China

**Keywords:** *Zizania latifolia*, mitochondrial genome, RNA editing, synteny, repeat sequence, phylogenetic, Oryzoideae

## Abstract

*Zizania latifolia* (Griseb.) Turcz. ex Stapf has been cultivated as a popular aquatic vegetable in China due to its important nutritional, medicinal, ecological, and economic values. The complete mitochondrial genome (mitogenome) of *Z. latifolia* has not been previously studied and reported, which has hindered its molecular systematics and understanding of evolutionary processes. Here, we assembled the complete mitogenome of *Z. latifolia* and performed a comprehensive analysis including genome organization, repetitive sequences, RNA editing event, intercellular gene transfer, phylogenetic analysis, and comparative mitogenome analysis. The mitogenome of *Z. latifolia* was estimated to have a circular molecule of 392,219 bp and 58 genes consisting of three rRNA genes, 20 tRNA genes, and 35 protein-coding genes (PCGs). There were 46 and 20 simple sequence repeats (SSRs) with different motifs identified from the mitogenome and chloroplast genome of *Z. latifolia*, respectively. Furthermore, 49 homologous fragments were observed to transfer from the chloroplast genome to the mitogenome of *Z. latifolia*, accounting for 47,500 bp, presenting 12.1% of the whole mitogenome. In addition, there were 11 gene-containing homologous regions between the mitogenome and chloroplast genome of *Z. latifolia*. Also, approximately 85% of fragments from the mitogenome were duplicated in the *Z. latifolia* nuclear genome. Selection pressure analysis revealed that most of the mitochondrial genes were highly conserved except for *ccmFc*, *ccmFn*, *matR*, *rps1*, and *rps3*. A total of 93 RNA editing sites were found in the PCGs of the mitogenome. *Z. latifolia* and *Oryza minuta* are the most closely related, as shown by collinear analysis and the phylogenetic analysis. We found that repeat sequences and foreign sequences in the mitogenomes of Oryzoideae plants were associated with genome rearrangements. In general, the availability of the *Z. latifolia* mitogenome will contribute valuable information to our understanding of the molecular and genomic aspects of *Zizania*.

## 1 Introduction

*Zizania*, a genus belonging to the Poaceae subfamily Oryza, comprises four species and is distributed between North America (*Zizania aquatica*, *Zizania palustris*, and *Zizania texana*) and Eastern Asia (*Zizania latifolia*) (Terrell et al., 1997). *Z. latifolia* as vegetable has been domesticated for more than 2,000 years and is predominantly cultivated in China, Vietnam, and other Asian countries. *Z. latifolia* was historically utilized as a significant staple food in ancient China and had been cultivated as an aquatic vegetable because its tender stems swell, soften, and becomes edible after being infected by the fungus *Ustilago esculenta* (Zhang et al., 2016).

At present, *Z. latifolia* has become the second largest aquatic vegetable after lotus root, and it is an important source of income for many farmers in southern China (Liu et al., 2022). Limited genomic information on *Zizania*, despite its diverse species and economic importance, hampers the effective utilization of genetic resources for research in phylogeny, genetics, and breeding. Several studies have conducted molecular phylogeny analyses of *Z. latifolia*, including the completed assembly of the chloroplast genome of *Z. latifolia*, providing insights into its genetic relationships. These studies contribute valuable information to our understanding of the molecular and genomic aspects of *Zizania* (Zhang et al., 2016; Yan et al., 2022). This limits the utilization of *Zizania* genetic resources for research of phylogeny, genetics, and breeding. However, to date, the assembled mitochondrial genome (mitogenome) of *Z. latifolia* has still not been reported to be assembled, while at the same time, its mitogenome characteristics have not been assessed.

It was not until 1992 that the first mitogenome of land plants was released, which greatly promoted basic research on mitogenome (Oda et al., 1992). Plant mitogenome sizes vary widely, from 66 kb in *Viscum scurruloideum* to over 11.3 Mb in *Silene conica* (Sloan et al., 2012; Skippington et al., 2015). However, within the Poaceae family, mitogenome size usually falls between 500 kb and 600 kb. Despite variations in size and structure, the number of genes in mitogenomes is relatively consistent, ranging from 14 to over 50 (Wang et al., 2024). Both protein-coding genes (PCGs) and non-coding regions exhibit variable lengths across mitogenomes (Kubo and Newton, 2008; Gualberto and Newton, 2017). There are 11,078 complete chloroplast genomes, and 1,613 plant mitochondrial genomes are available on the National Center for Biotechnology Information (NCBI) (https://www.ncbi.nlm.nih.gov/genome/browse#!/overview/, accessed on January 12, 2024), which provides a basis for the study of mitogenomes.

Mitochondrial genomes of various Poaceae species, such as *Oryza minuta*, *Saccharum* spp., *Sporobolus alterniflorus*, *Avena longiglumis*, *Elymus magellanicus*, and *Eleusine indica*, have been published. These genomes serve as crucial resources for conducting comparative mitogenomic studies within the Poaceae family (Asaf et al., 2016; Chen et al., 2012; Hall et al., 2020; Wang et al., 2021; Li et al., 2022; Liu et al., 2023; Chen et al., 2023). Mitogenomes in the Poaceae family typically comprise a single circular DNA molecule, as observed in *O. minuta*, *S. alterniflorus*, *A. longiglumis*, *E. magellanicus*, and *E. indica*. However, *Saccharum* spp. stand out as an exception, having two distinct circular DNA molecules in their mitogenome (Notsu et al., 2002). As a Poaceae species closely related to rice (*Oryza sativa*), *Z. latifolia* has many excellent traits that cultivated rice lacks, such as high protein, high biomass, tolerance to deep water, resistance to rice blast, and abnormally fast grain filling speed. It can serve as an excellent gene donor material, offering valuable genetic resources for rice breeding purposes (Hirabayashi et al., 2015; Neelam et al., 2017; Wu et al., 2018; Zhong et al., 2019; Yan et al., 2022).

However, the mitogenome of *Z. latifolia* remains unpublished, and its numerous properties are yet to be uncovered. In this research, the complete mitogenome sequence of *Z. latifolia* was *de novo* assembled using a combination of second-generation Illumina sequencing and third-generation PacBio sequencing technologies, and then the mitogenome organization, characteristic, phylogenetic relationship, and comparative genome analyses were performed. This study aims to establish a theoretical foundation for omics research, biological functions, genome evolution, and genetic breeding of *Z. latifolia* while also contributing to a comprehensive understanding of the structural characteristics and evolutionary diversity of the mitogenome within the Poaceae family.

## 2 Materials and methods

### 2.1 Plant materials and genome sequencing

In this study, *Z. latifolia* ‘Dabieshan No.1’ plants were grown and collected in the greenhouse of Anhui Agricultural University (31°86′N, 117°25′E). Fresh young leaves of *Z. latifolia* were quickly frozen in liquid nitrogen and stored in a −80°C refrigerator. Illumina paired-end reads were generated using the Illumina NovaSeq platform, and HiFi reads were generated using the PacBio Sequel platform.

### 2.2 Mitogenome assembly and annotation

A hybrid assembly strategy was used for *Z. latifolia* mitogenome assembly. First, the GetOrganelle v1.7.6.1 (Jin et al., 2020) software was used to assemble the mitogenome short reads and assemble those reads into a corresponding unitig graph with the parameters ‘-R 30 -k 65,85,105,115,127 -F embplant_mt’, and the contigs that contained the mitochondrial core genes in Poaceae were selected. Then, the PacBio HiFi reads were *de novo* assembled by flye (Kolmogorov et al., 2019) 2.9.1-b1780 with the parameters ‘–pacbio-corr –meta -g 500K -t 20’, and the final mitogenome was visualized and adjusted manually by the Bandage (Wick et al., 2015) software.

The mitogenome of *Z. latifolia* was annotated by Geseq (https://chlorobox.mpimp-golm.mpg.de/geseq.html) and IPMGA (http://www.1kmpg.cn/mgavas/) with reference genomes, including *Oryza rufipogon* (NC_013816.1), *O. minuta* (NC_029816.1), *Oryza coarctata* (MG429050.1), and *O. sativa* (JF281153.1), and then manually corrected. The mitogenome structure map was drawn using OGDRAW (http://ogdraw.mpimp-golm.mpg.de/cgi-bin/ogdraw.pl). The assembled mitogenome sequence of *Z. latifolia* has been submitted to GenBank.

### 2.3 Repeat sequence analysis

Simple sequence repeats (SSRs) using MISA (MIcroSAtellite) perl script for SSR analysis (Beier et al., 2017) and parameters were set as follows (unit_size, min_repeats): 1–10 2–5 3–5 4–4 5–3 6–3. Tandem repeat finder v4.09 software with default parameters (http://tandem.bu.edu/trf/trf.submit.options.html) detects tandem repeats (>6-bp repeats). Furthermore, reverse, forward, palindromic, and complementary repeat sequences were identified using REPuter (https://bibiserv.cebitec.uni-bielefeld.de/reputer/) with the default settings.

### 2.4 Chloroplast to mitochondrion DNA transformation and nuclear mitochondrial DNA segments

To investigate the fragment transfer between the mitogenome of *Z. latifolia*, chloroplast genome, and *Z. latifolia* nuclear genome, BLASTN 2.5.0+ was utilized to identify the transfer fragments between mitogenome with chloroplast genome (MTPTs), mitogenome with the nuclear genome (NUMTs), and chloroplast genome with chloroplast genome (NUPTs), and parameters were set as follows: -evalue 1e^-10^ -outfmt 6. The Circos diagram was visualized using the Advanced Circos module in Tbtools (Chen et al., 2023). The density distribution map of NUMTs and NUPTs was visualized using the R package CMplot.

### 2.5 Transcriptome sequencing and RNA editing event

For transcriptome sequencing, total RNA was extracted, the DNA was digested with DNase, and then the mRNA of eukaryotic organisms was enriched with magnetic beads with Oligo (dT). The mRNA was broken into short fragments by adding an interrupting reagent, and then one-strand cDNA was synthesized using a random 6-bp primer with the interrupted mRNA as a template. Then, a two-stranded reaction system was formulated to synthesize two-stranded cDNA, and the kit was used to purify double-stranded cDNA; for end repair, A-tail was added, the sequencing junction was connected, the fragment size was selected, and finally PCR amplification was conducted. Constructed libraries were qualified using Agilent 2100 Bioanalyzer and then used to amplify mRNA using the Illumina HiSeq™. After the constructed library was qualified using Agilent 2100 Bioanalyzer, Illumina HiSeq™ 2500 was used to generate the double-ended data of 150 bp. After passing QC, sequencing was performed using an Illumina sequencer.

The identification process of RNA editing sites can be decomposed into three steps: first, read alignment; second, the variant calling; and third, detection of RNA editing sites. For each species, to increase sequencing depth, all the replicates were merged into one sample. The quality control of paired-end Illumina sequencing data was evaluated first by fastp (Chen et al., 2018) in the default parameter. Clean reads were mapped to reference (*Z. latifolia* mitogenome and chloroplast genome assembled in this study) using hisat2 software under default parameters (Kim et al., 2019). Afterward, the alignment results were sorted, duplicates were removed, and SAMtools was used for indexing (Li et al., 2009). Finally, the resulting BAM file was then used to call variants using bcftools, and VCF files that describe transcriptome variation were generated (Danecek and McCarthy, 2017). For each species, based on the variant-calling results (in “VCF” format) and genome annotation files (in “tbl” format), RNA editing sites were identified under default parameter values using the REDO tool (Wu et al., 2018).

### 2.6 Codon preference analysis

The PCG sequence of *Z. latifolia* mitogenome was extracted using PhyloSuite v1.1.12 (Zhang et al., 2020). MEGA-X was applied to analyze the relative synonymous codon usage (RSCU) of PCGs and calculate the RSCU value (Kumar et al., 2016). A codon with RSCU > 1 indicated a preference for amino acid usage, RSCU = 1 implied no preference, and RSCU < 1 indicated contrary codon usage.

### 2.7 Non-synonymous and synonymous substitution analysis

The non-synonymous (Ka) and synonymous (Ks) substitution (Ka/Ks) was analyzed for shared PCGs between *Z. latifolia* and six other species, including *O. rufipogon* (NC_013816.1), *O. sativa* (JF281153.1), *O. minuta* (NC_029816.1), *O. coarctata* (MG429050.1), *Zea perennis* (NC_008331.1), and *Triticum aestivum* (NC_036024.1). MAFFT (v7.313) was used to align all PCG sequences, and Ka/Ks calculator version 2.0 was utilized for Ka/Ks analysis (Wang et al., 2010).

### 2.8 Phylogenetic analysis

To better and more comprehensively explore the evolutionary relationship of *Z. latifolia*, the mitogenome data of 30 higher plants (**Supplementary Table 8**) were downloaded from the NCBI Organelle Genome Database. A total of 31 shared PCGs among the analyzed species were identified and extracted using PhyloSuite (v.1.2.2) (Zhang et al., 2020). All the genes were aligned in batches using MAFFT (v7.313) integrated into PhyloSuite using normal-alignment mode. Maximum likelihood phylogenies were inferred using IQ-TREE under Edge-unlinked partition model for 50,000 ultrafast bootstraps, and the trees were visualized using iTOL. To ascertain the taxonomic status of the mitogenome in *Z. latifolia*, phylogenetic analysis was carried out with 30 other species, including 26 eudicotyledons, 13 monocotyledon plants, and one gymnosperm plant, with *Amborella trichopoda* as outgroup (**Supplementary Table 8**).

### 2.9 Collinear analysis and comparative genome analysis

Six species closely related to *Z. latifolia* were selected for collinear analysis, including *O. rufipogon* (NC_013816.1), *O. sativa* (JF281153.1), *O. minuta* (NC_029816.1), *O. coarctata* (MG429050.1), *Z. perennis* (NC_008331.1), and *T. aestivum* (NC_036024.1), to conduct mitogenome rearrangement, and collinearity of *Z. latifolia* was compared using the Mauve programs.

To investigate the characteristics of the mitogenomes of *Z. latifolia* and other species (**Supplementary Table S8**), we conducted an analysis of the size and guanine–cytosine (GC) content of the mitogenomes. The species used for comparative analysis included gymnosperms and angiosperms in key positions of mitochondrial phylogenetic evolution (Ye et al., 2017).

## 3 Results

### 3.1 Mitogenome assembly, annotation, and gene features

**Figure 1:**
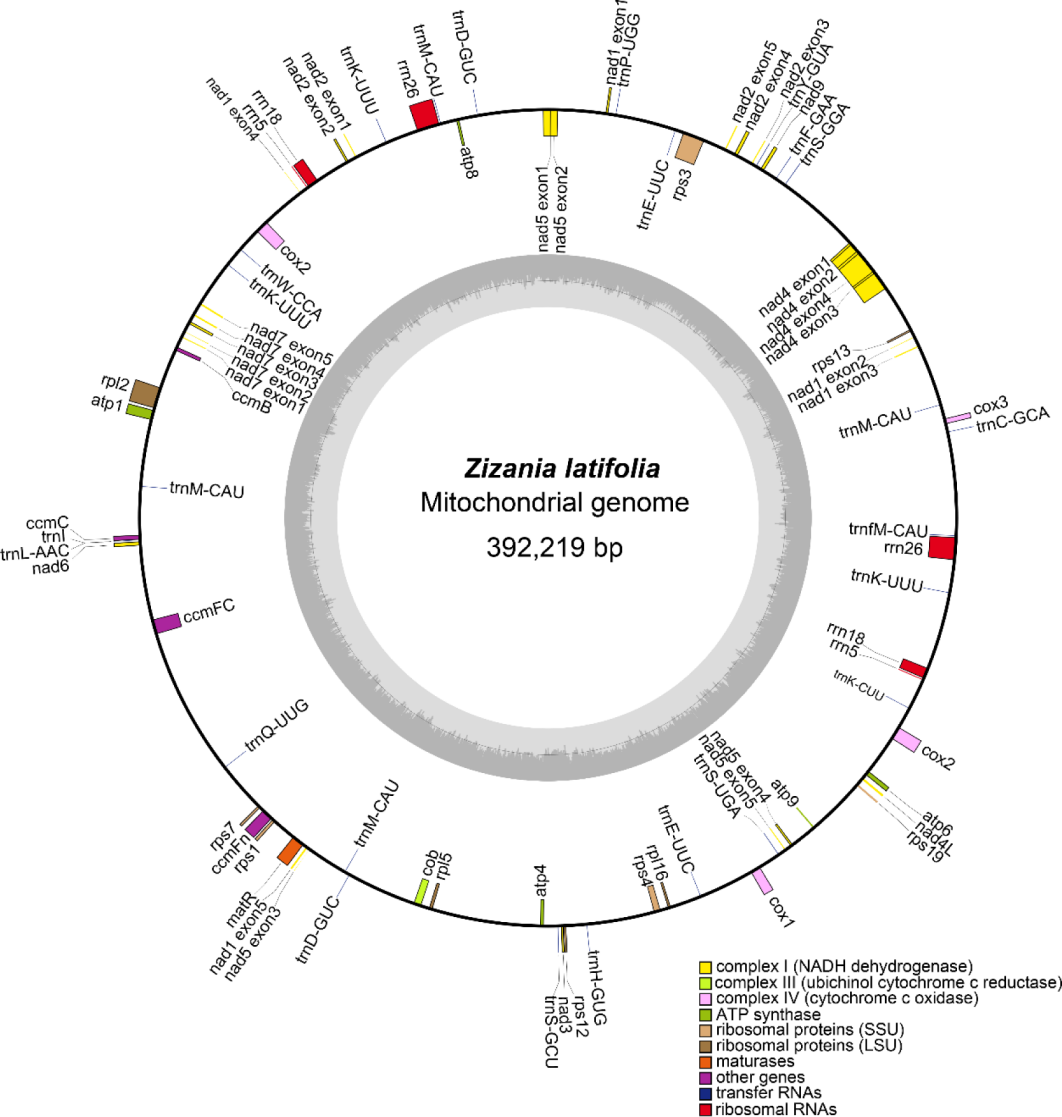
*Zizania latifolia* mitogenome circular map.

Accurate *Z. latifolia* mitogenomes were obtained by combining Illumina and PacBio reads. Consistent depths of mapping reads revealed the high-quality gap-free assembly (**Supplementary Table 1**). The mitogenome of *Z. latifolia* was assembled into a single circular molecule, with a total length of 392,219 bp, and *Z. latifolia* mitogenome had a nucleotide makeup of A (27.75%), T (27.62%), G (22.20%), and C (22.43%) and a GC content of 44.63% (**Figure 1**). A total of 49 unique genes were annotated, including 33 PCGs, 13 tRNA genes, and three rRNA genes, and identified in the *Z. latifolia* mitogenome. Protein-coding genes accounted for 7.96% of the whole mitogenome, while tRNA and rRNA genes comprised only 0.56% and 1.71%, respectively. Interestingly, it was found that the *cox2* gene had two copies. In addition, three rRNA and five tRNA genes also had two to five copies (*rrn5*, *rrn26*, *rrn18*, *trnN-GTT*, *trnM-CAT*, *trnP-TGG*, *trnH-GTG*, *trnW-CCA*, and *rrn5*). The annotated genes in *Z. latifolia* contained introns, and six of those genes (*rps2*, *rps3*, *nad5*, *cox2*(2), *rpl2*, and *ccmFc*) included an intron (**Table 1**).

**Table 1:** Gene profile and organization in the mitogenome of *Zizania latifolia*.

Types of genes	Gene name
ATP synthase	*atp1*, *atp4*, *atp6*, *atp8*, *atp9*
Cytochrome *c* biogenesis	*ccmB*, *ccmC*, *ccmFn*, ccmFC*
Cytochrome *c* oxidase	*cox1*, *cox2* (2) *, *cox3*
NADH dehydrogenase	nad1****, nad2****, nad3,nad4***, *nad4L*, nad5****, *nad6*, nad7****, *nad9*
Maturases	*mat-r*
Ubiquinol cytochrome *c* reductase	*Cob*
Small subunit of ribosome	*rps1*, rps3*, *rps4*, *rps7*, *rps12*, *rps13*, *rps19*
Large subunit of ribosome	*rpl16*, rpl2*, *rpl5*
Ribosomal RNAs	*rrn5* (2), *rrn26* (2), *rrn18* (2)
Transfer RNAs	*trnC*, *trnD* (2), *trnE* (2), *trnF*, *trnH*, *trnI*, *trnK* (4), *trnL*, *trnM* (5), *trnN*, *trnP*, *trnQ*, *trnS*(3), *trnW*, *trnY*

Gene(2): Number of copies of multi-copy genes. * for introns, the number of * for the number of introns.

### 3.2 Repeat sequences, codon usage, and RNA editing site analysis

**Figure 2:**
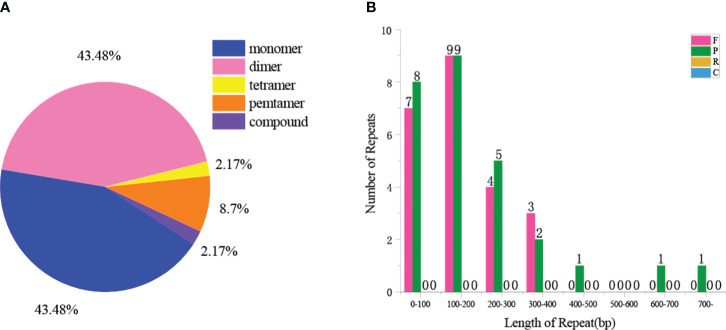
Repeat sequences in the mitogenome of *Zizania latifolia*. **(A)** Type and proportion of simple sequence repeats (SSRs). **(B)** The length distribution of dispersed repeat.

A total of 46 SSRs were found in the *Z. latifolia* mitogenome, and SSRs in monomeric and dimeric forms accounted for 87.0% of the total SSRs (**Figure 2**). Adenine (A) monomeric repeats accounted for 45% of *Z. latifolia* monomeric SSRs, and TA repeats were the most common type of dimeric SSRs, accounting for 30% (**Figure 2A**). The above SSRs can be used as potential markers to identify *Z. latifolia* (**Supplementary Table 2**). A total of 36 tandem repeats with a match of more than 70% and a length between 26 and 149 bp were found in the *Z. latifolia* mitogenome (**Supplementary Table 3**). A total of 50 dispersed repeat sequences with lengths greater than or equal to 80 were observed, including 23 pairs of direct repeats and 27 pairs of reverse repeats (**Figure 2B**), of which the longest direct repeat was 38,743 bp.

**Figure 3:**
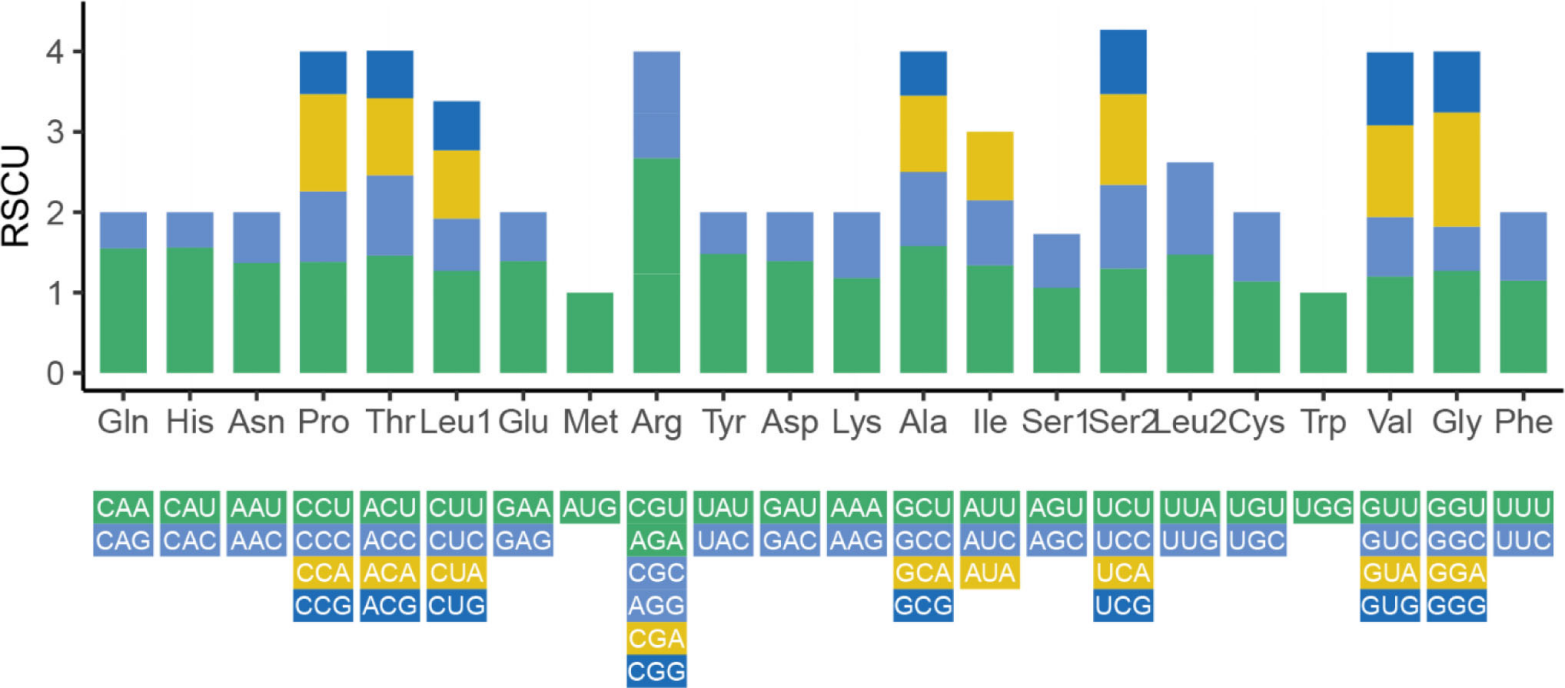
Relative synonymous codon usage (RSCU) of *Zizania latifolia* mitogenome.

We also analyzed the RSCU of 35 PCGs in the *Z. latifolia* mitogenome (**Figure 3**). The codon usage analysis revealed the most frequent amino acids to be leucine (Leu) (10.51%), serine (Ser) (8.95%), and isoleucine (lle) (7.6%), while cysteine (Cys) and tryptophan (Trp) were rarely found (**Supplementary Table 4**). Codon usage was generally strongly biased toward A or T(U) at the third codon position in the *Z. latifolia* mitogenome, which was commonly found in the mitogenomes of land plants (Li et al., 2023). A total of 25 shared codons encoding 16 amino acids (one was a stop codon, UGA) showed that RSCU exceeded 1 in the mitogenome of *Z. latifolia*. In addition to the RSCU value of the initial codon AUG and tryptophan (UGG), there is a common codon preference for PCGs in mitochondria (Li et al., 2023).

**Figure 4:**
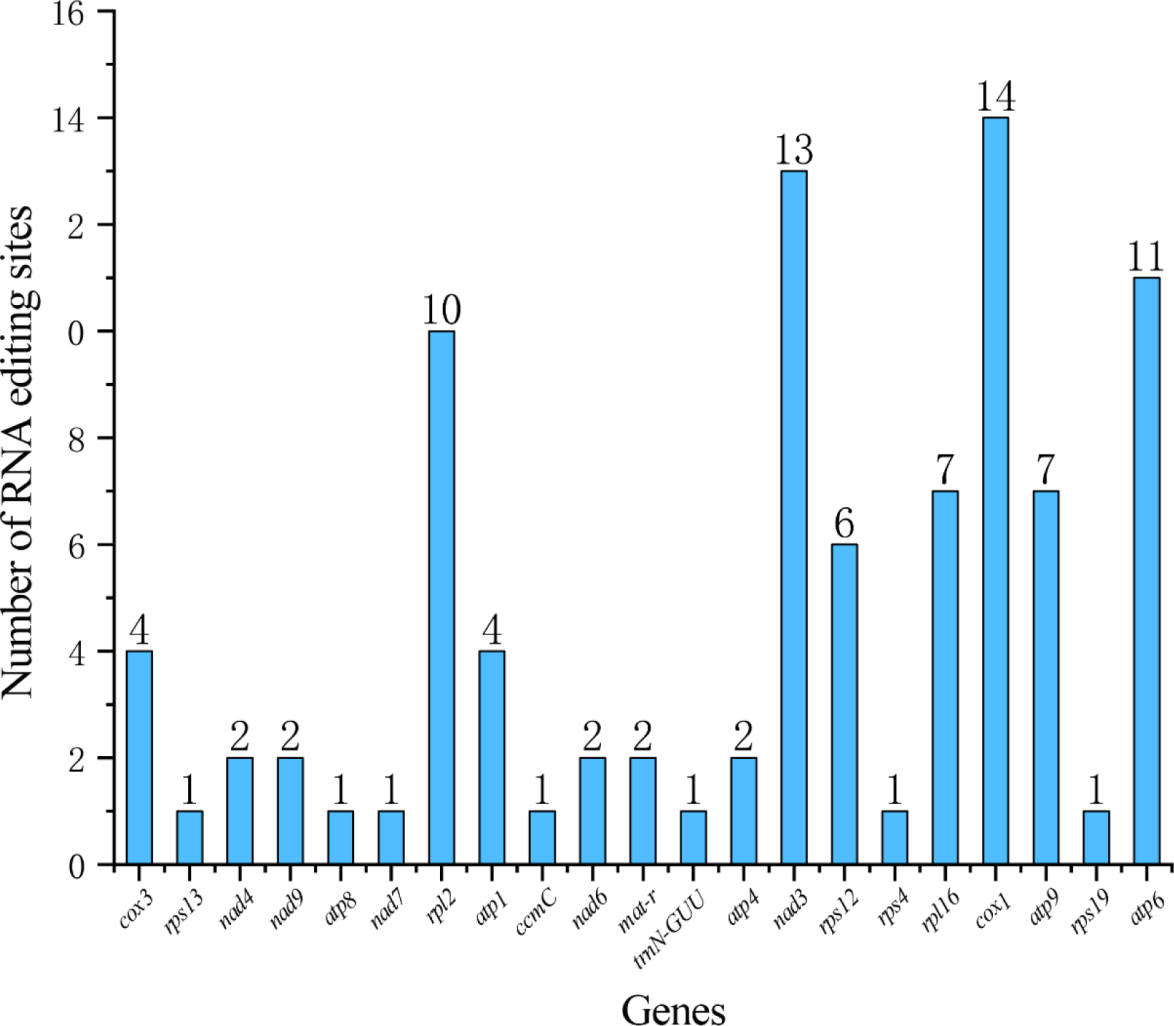
The distribution of RNA editing sites in 21 genes of the mitogenome of *Zizania latifolia*.

A total of 93 RNA editing sites were identified on 21 genes in the *Z. latifolia* mitogenome (**Supplementary Table 5**). The *cox1* gene identified 14 RNA editing sites, far more than other genes (**Figure 4**). Furthermore, we identified a total of six different types of RNA editing, and C to T editing was the most common in mitogenome. Most RNA editing events caused serine (Ser) and proline (Pro) to be replaced with leucine (Leu) during translation, with 18 and 11 occurrences, respectively, which accounted for 19.13% and 11.8% of the total number of identified events (**Supplementary Table 5**). Additionally, 51.1% of amino acids remained hydrophobic, 36.9% became hydrophobic, and 6.5% became hydrophilic (**Supplementary Table 6**).

### 3.3 Chloroplast-derived and nuclear-derived sequence analysis

**Figure 5:**
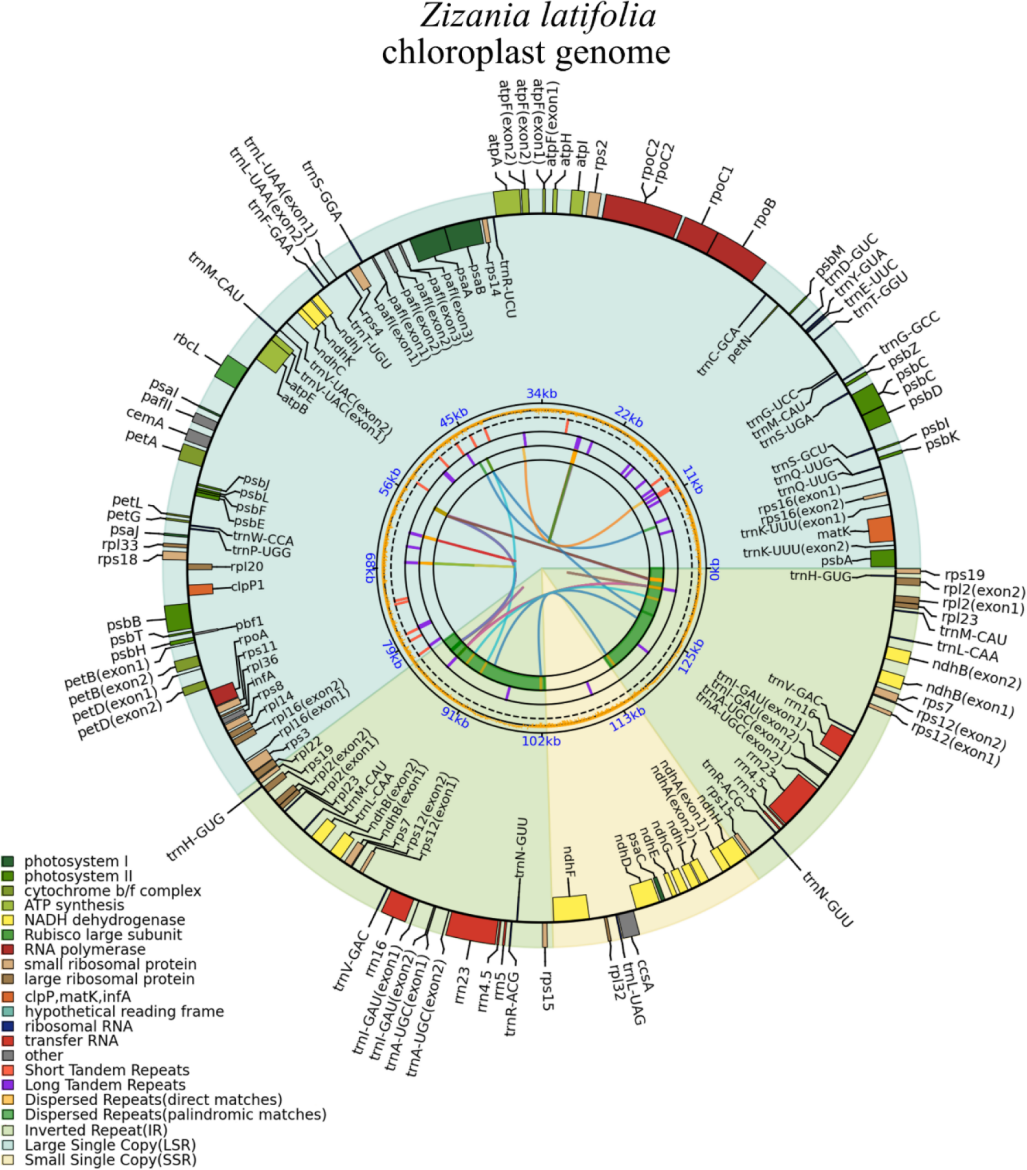
Gene map of the *Zizania latifolia* chloroplast genome.

Throughout the evolution of mitochondria, fragments from chloroplasts have transferred into the mitogenome. In the mitogenome, approximately 5%–10% of sequences can be identified as homologous sequences derived from the chloroplast genome (Sloan and Wu, 2014; Petersen et al., 2020). In this research, the chloroplast genome of *Z. latifolia* was reassembled and annotated (**Figure 5**), followed by a comparative analysis of the mitochondrial and chloroplast genomes.

**Figure 6:**
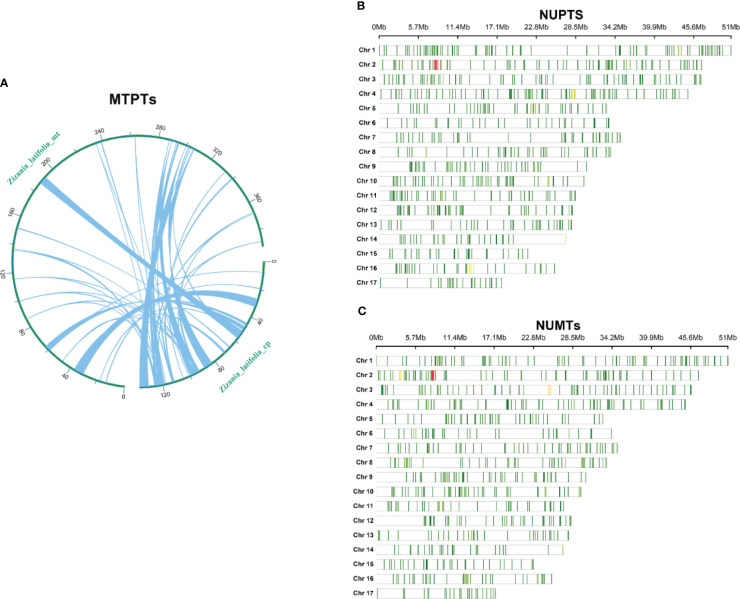
Chloroplast-derived and nuclear-derived sequences in *Zizania latifolia*. **(A)** Schematic representation of transfers between chloroplast and mitogenome (MTPTs) of *Z. latifolia*. **(B)** Distribution of the sequences homologous with nuclear genome in chloroplast genome (MUPTs) in *Z. latifolia*. **(C)** Distribution of the sequences homologous with nuclear genome (NUMTs) in mitogenome in *Z. latifolia*.

It was determined that the mitogenome sequence of *Z. latifolia* (392,219 bp) was approximately 2.87 times longer than the chloroplast genome (136,503 bp). Homologous fragment transfer analysis was performed on *Z. latifolia* mitochondrial and chloroplast genome segments (MTPTs) (**Figure 6A**). A total of 49 homologous fragments, comprising 47,500 bp, were identified between the mitogenome and chloroplast genomes, representing respectively 12.1% and 34.7% of their respective genome lengths (**Supplementary Table 7**). The longest fragment was 6,226 bp, and the shortest fragment was 30 bp. Eleven gene-containing homologous segments were identified between the chloroplast and mitogenome of *Z. latifolia*, including nine chloroplast genes: *trnC-GCA*, *trnC-CAU*, *trnA-GGA*, t*rnF-GAA*, t*rnW-CCA*, *trnW-CAU*, *trnW-GUU*, *trnH-GUG*, and *rrn18* (fragment).

Investigating the genetic interactions between the chloroplast genome, mitogenome, and nuclear genome of *Z. latifolia* revealed extensive intracellular gene transfer (**Figure 6**). A total of 1,752 sequences from the chloroplast genome, spanning approximately 395 kb, were identified in the nuclear genome. These sequences varied in length from 43 to 10,719 bp, averaging 225 bp. The distribution of homologous fragments in the nuclear genome was uneven across 17 chromosomes of *Z. latifolia*, with chromosome 2 exhibiting the highest homologous sequences (2,263 fragments) and chromosome 15 the least (44 fragments) (**Figure 6B**).

Similarly, approximately 335 kb of sequences from the *Z. latifolia* mitogenome showed homology with the nuclear genome. In the nuclear genome, the most homologous sequences were found on chromosome 2 (326 fragments), whereas the least homologous sequences were found on chromosome 15 (50 fragments). A total of 2,047 fragments from the mitogenome were identified in the nuclear genome, with lengths ranging from 44 to 9,400 bp and an average length of 164 bp. These findings suggested significant genetic transfer between the nuclear genome and the chloroplasts and mitogenomes during the evolution of *Z. latifolia*.

### 3.4 Collinearity analysis and gene rearrangement

**Figure 7:**
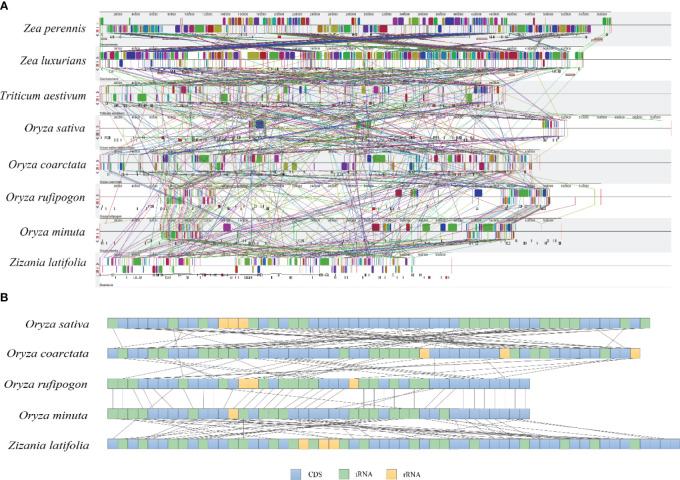
Collinearity and gene rearrangement analysis of *Zizania latifolia*. **(A)** Mauve alignments of *Z. latifolia* mitogenome with seven closed species. **(B)** The gene arrangement of mitogenomes in Oryzoideae; the boxes under corresponding mitogenome refer to annotated genes, and the boxes refer to CDS, tRNA, or rRNA.

To further investigate the structural variation and collinearity, the mitogenome of *Z. latifolia* and other seven Poaceae species were compared using Mauve (**Figure 7A**). The results of the covariance indicated that the species in the Oryzoideae subfamily had more homologous regions with *Z. latifolia*. The contraction or expansion of homologous sequences of different sizes and numbers resulted in dynamic changes in the mitogenome of *Z. latifolia*. Many homologous collinear blocks were detected between *Z. latifolia* and *O. minuta*, indicating that the more closely related species retained more collinear blocks. These homologous regions had different relative positions, indicating abundant rearrangements within those mitogenomes, which were also related to the differences in the relative order of genes in the Oryzoideae species (**Figure 7B**).

### 3.5 Phylogenetic analysis

The mitogenomes of 29 species were downloaded from the NCBI dataset to determine the evolutionary position of the *Z. latifolia* mitogenome. *A. trichopoda*, a basal angiosperm, and *Ginkgo biloba*, a representative species of gymnosperms, were used as outgroups in phylogenetic analysis. *Vitis vinifera* and *Arabidopsis thaliana*, representative species of dicotyledons, were chosen as representative species. The other 25 species were all monocotyledons, 21 of which were from the Poaceae. Phylogenetic analyses were performed using the maximum likelihood method after concatenation and alignment based on 11 conserved mitochondrial PCGs shared in 30 mitogenomes (*atp9*, *ccmC*, *cox1*, *cox2*, *cox3*, *cytb*, *nad3*, *nad6*, *nad9*, *rps7*, and *rps12*).

**Figure 8:**
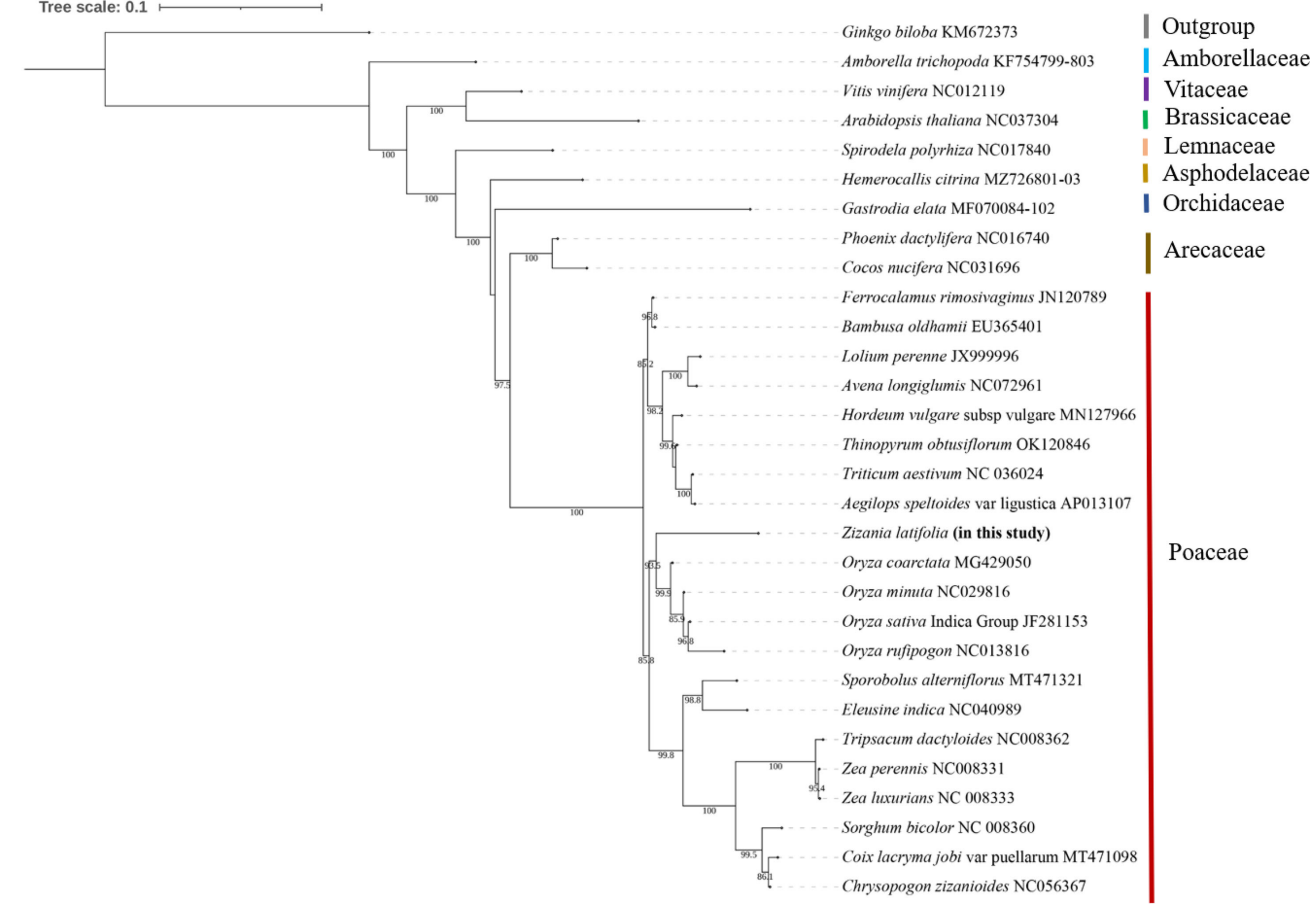
Phylogenetic tree of 30 species. Outgroup: *Ginkgo biloba* and *Amborella trichopoda*.

The phylogenetic analysis of 30 plant mitogenomes revealed their classification into nine major groups: Ginkgoaceae, Amborellaceae, Vitaceae, Brassicaceae, Lemnaceae, Asphodelaceae, Orchidaceae, Arecaceae, and Poaceae (**Figure 8**). Within the Poaceae clade, five species formed a distinct cluster, with *Z. latifolia* showing the closest relationship to *O. coarctata*. This alignment was consistent with conclusions drawn from the nuclear genome. Notably, *Z. latifolia* occupied a position closer to the root of the phylogenetic tree, suggesting an earlier differentiation in the evolution of Oryzoideae. The topological structure of the phylogeny, based on 11 conserved mitochondrial protein-coding genes, is consistent with the classification outlined by the Angiosperm Phylogeny Group (APG IV) (The A. P. G et al., 2016).

### 3.6 Nucleotide diversity analysis and the synonymous and non-synonymous substitution

**Figure 9:**
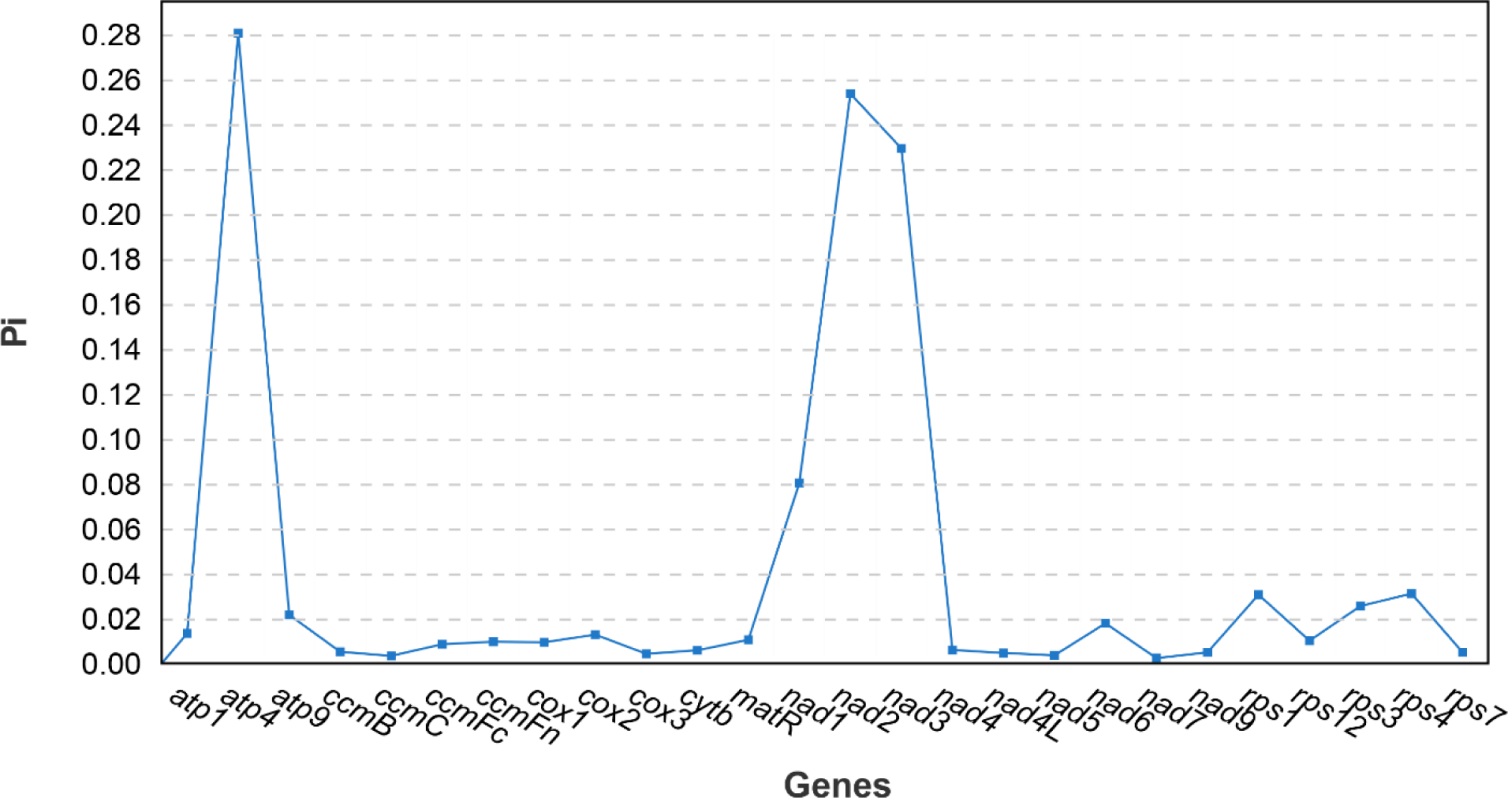
Nucleotide diversity (Pi) analysis of 26 protein-coding genes (PCGs) in the mitogenome of *Zizania latifolia* and seven species (*Oryza sativa*, *Oryza coarctata*, *Zea perennis*, *Zea luxurians*, *Oryza rufipogon*, *Oryza minuta*, and *Triticum aestivum*).

The nucleotide diversity of 26 PCGs in the mitogenome of *Z. latifolia* and seven species (*O. sativa*, *O. coarctata*, *Z. perennis*, *Zea luxurians*, *O. rufipogon*, *O. minuta*, and *T. aestivum*) was calculated to assess the level of sequence divergence in these species. There were eight hypervariable gene regions with Pi values greater than 0.02: *atp4* (0.28), *atp9* (0.021), *nad1* (0.080), *nad2* (0.25), *nad3* (0.23), *rps1* (0.031), *rps3* (0.026), and *rps4* (0.031) (**Figure 9**). There were eight PCGs in the mitogenome *Z. latifolia*, three of which (*atp4*, *nad2*, and *nad3*) had Pi values exceeding 0.1000. These divergence hotspot regions of *Z. latifolia* mitogenome could be developed as effective DNA markers for phylogenetic analyses and species identification in the Poaceae family.

**Figure 10:**
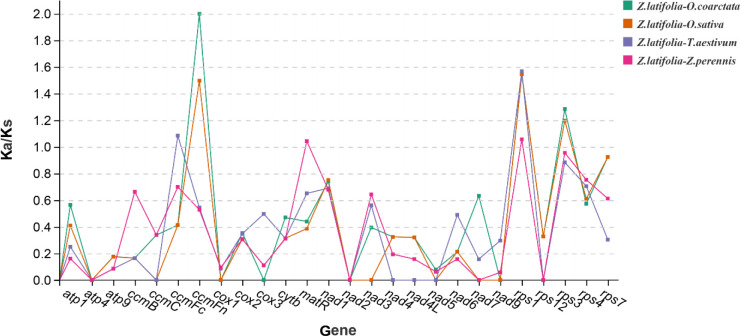
Non-synonymous and synonymous substitution (Ka/Ks) of 26 protein-coding genes (PCGs) in *Zizania latifolia*, *Oryza coarctata*, *Triticum aestivum*, *Oryza sativa*, and *Zea perennis*.

The Ka and Ks nucleotide substitution plays an indispensable role in molecular evolution (Shtolz and Mishmar, 2019; Zhang et al., 2006). To explore the evolutionary rates of mitochondrial genes, we computed and compared the Ka/Ks values of protein-coding genes shared in five mitotic genomes (**Figure 10**). Ka/Ks values for the five shared PCGs between *Z. latifolia* and *O. coarctata* were zero. For the eight shared PCGs between *Z. latifolia* and *O. sativa*, the Ka/Ks = 0. The Ka/Ks ratio of *rps3*, *rps1*, and *ccmFn* genes in *Z. latifolia* compared to *O. coarctata* and *O. sativa* was larger than 1, indicating that those genes were under positive selection in its evolution. However, most of the PCGs with Ka/Ks < 1 indicated that negative selection effects occurred in the genes of *Z. latifolia* compared to the other four species. This finding suggests that most of the PCGs in the mitogenome of *Z. latifolia* were highly conserved during molecular evolution and may play a key role in cellular activities such as respiration.

### 3.7 Gene duplication and loss, and characteristic difference of mitogenomes

Gene duplication and loss often occur during the evolution of plants, and the genes that are retained by duplication are crucial for normal life activities. Therefore, gene duplication and loss in 30 species were compared here, and a total of 73 gene duplication and gene loss had been identified (**Supplementary Figure 1**). In mitochondrial PCGs shared in 30 species, no loss was found of ATP synthase genes and cytochrome *c* synthesis genes, which were strongly conserved. However, the succinate dehydrogenase protein (*sdh3* and *sdh4*) and tRNA were lost during the evolution of the Poaceae and were highly variable and weakly conserved.

**Figure 11:**
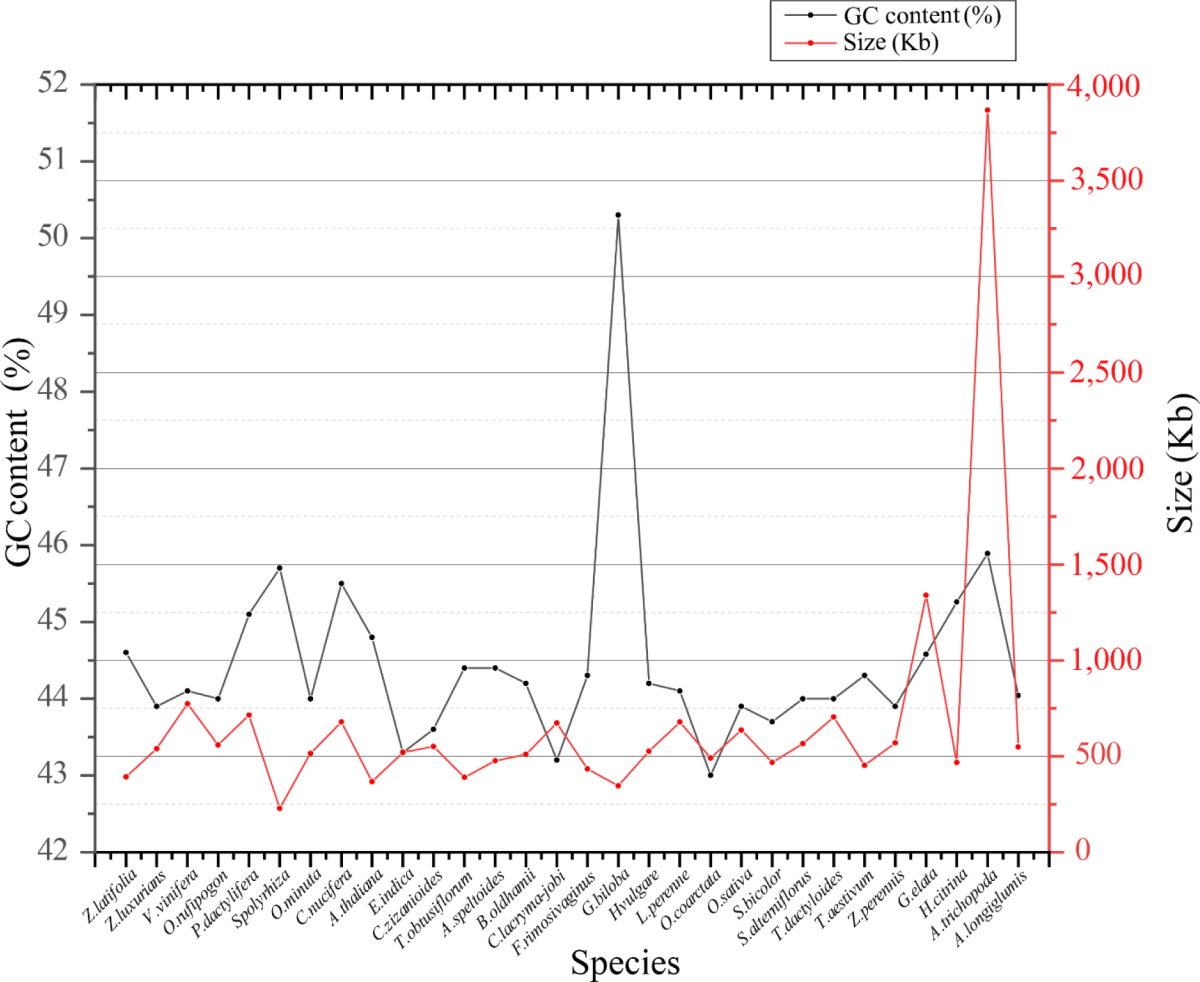
Gene content (GC %) and genome size (kb) of 30 plant mitochondrial genomes.

In the analysis of genetic characteristic differences in *Z. latifolia*, we conducted a comparative study on the size and GC content of mitogenomes across 30 species (Li et al., 2023) (**Supplementary Table 8**). Among Poaceae, mitochondrial sizes exhibited relatively small differences, ranging from 390,725 bp (*Thinopyrum obtusiflorum*) to 548,455 bp (*A. longiglumis*). The Poaceae mitogenome demonstrated overall stability during evolution, with GC content varying from 43% (*O. coarctata*) to 44.6% (*Z. latifolia*) (**Figure 11**). Although the size of Poaceae mitogenomes varied, the consistent GC content suggested that the evolution of mitogenomes had been consistently robust.

## 4 Discussion

### 4.1 Characterization of the *Z. latifolia* mitogenome

In this study, the mitogenome of *Z. latifolia* was assembled and annotated, revealing a closed single circular structure spanning 321,219 bp. In addition, the GC content of the mitogenome of *Z. latifolia* was 44.63%, which was comparable to other close-related mitogenomes (for example, *O. sativa* Indica Group, 43.9%). At present, the mitogenomes of *Z. latifolia* (reported in this study), *O. sativa* (Notsu et al., 2002), and other closely related genera in Oryzoideae (Asaf et al., 2016) all have a single ring structure. Mitogenomes for the five Oryzoideae species ranged in size from 392,219 bp to 637,692 bp.

RNA editing is a common phenomenon in mitogenomes and is necessary to maintain genetic information and normal function of mitochondrial gene RNA levels, which are associated with important plant cultivation traits (Bi et al., 2016; Sun et al., 2016; Yang et al., 2017; Peng et al., 2018). We detected 93 RNA editing sites in the mitogenome of *Z. latifolia*. After RNA editing, the hydrophobicity properties of 43.74% of the amino acids did not change, and 55.51% of the amino acids changed. The number of RNA editing events detected in *cox1*, *nad3*, and *atp6* was 14, 13, and 11, respectively. They are important components of the mitochondrial respiratory chain and play an important role in adapting to aquatic environments and coping with environmental stresses. The identification of these RNA editing sites provides necessary clues for future exploration of evolution and novel codon prediction gene function and contributes to our better understanding of gene expression in plant mitogenomes. Codon usage was generally strongly biased toward A or T(U) at the third codon position in the *Z. latifolia* mitogenome. This finding was consistent with the results of previous studies on *T. aestivum* (Zhang et al., 2007) and *Zea mays* (Zhou and Li, 2009).

Chloroplast-derived sequences are ubiquitously present in all currently sequenced plant mitogenomes (Hong et al., 2021). The MTPT of rice accounts for 6.20% of the mitogenome; the plastid homologous sequence found in *A. longiglumis* is relatively small, only 8,207 bp, accounting for 1.5% of the mitogenome. Our study found 28,207 bp of homologous sequences between the mitogenome and chloroplast genome of *Z. latifolia*, which was approximately 7.80% of the total length of the mitogenome. The transfer segments may influence the expansion of the mitogenome size. In this study, annotation of chloroplast-derived sequences revealed that most of the chloroplast-derived sequences were homologous short segments of non-coding regions in chloroplasts, but a few were annotated as tRNA. Chloroplast-derived tRNA in mitochondria may be complementary to tRNA in the mitochondrial genome.

In addition, the transfer of DNA fragments between organelle genomes and nuclear genomes is also an important event in plant evolution (Qu et al., 2023). We found many small-size NUMTs distributed over all nuclear chromosomes, which was similar to the results obtained for *A. longiglumis* (the diploid oat) and *Populus deltoides* (Liu et al., 2023; Qu et al., 2023). These fragments may play an important role in evolution. For example, the rate of transfer of chloroplast DNA to the nucleus in tobacco may have a significant impact on existing nuclear genes (Huang et al., 2003). By analyzing the homologous sequences between the chloroplasts and mitogenomes and the nuclear genome, we can infer the exchange of genetic material between gene organelles and the nucleus, thereby providing genomic data for research on nucleocytoplasmic interactions in *Z. latifolia*.

### 4.2 Repeat sequences and nucleotide diversity analysis

The mitogenome contains many repetitive sequences, encompassing tandem repeats, short repeats, and long repeats. These sequences constitute a predominant component of non-coding regions, influencing both functional gene domains and mitochondrial attributes, thereby impacting mitochondrial function and biological characteristics (Dong et al., 2018). In this study, dispersed repeat sequences and simple repeat sequences of the *Z. latifolia* mitogenome accounted for 9.44% and 8.93% of the total length, respectively. Repetitive sequences account for approximately 12.9% of the mitogenome of *Z. latifolia*. Among them, there were 87 repeat sequences with a length of approximately 1 kb, and the longest repeat sequence was approximately 5.5 kb. The wide distribution of these sequences may be the reason for the increase in size of the *Z. latifolia* mitogenome.

Individual plants with unique genotypes can be clearly identified by utilizing molecular markers, which have been applied in *Amorphophallus albus* and *O. sativa* (Beser et al., 2021; Shan et al., 2023). Prior investigations have meticulously examined the genetic diversity and genetic structure of *Z. latifolia* species resources using SSR markers (Xu et al., 2008; Quan et al., 2009; Millar et al., 2011; Chen et al., 2012). This analytical approach has significantly advanced the classification and genetic scrutiny of *Z. latifolia*. Currently, the limited quantity of SSR markers in *Z. latifolia*, combined with its singular genomic coverage in the mitogenome, emphasizes the potential significance of identifying novel SSR markers. The identification of novel SSR markers within the mitogenome holds promise as a foundational advancement, poised to facilitate extended investigations into the genetic diversity and structure of wild *Zizania* resources.

Nucleotide diversity serves as a metric to elucidate variations in nucleic acid sequences among different species, with regions exhibiting higher variation potentially offering valuable molecular markers for population genetics (Tong et al., 2017; Li et al., 2022). In the mitogenome of *Z. latifolia*, we identified 11 genes through comparative analysis, revealing Pi values ranging from 0.00063 to 0.02182, with most Pi values falling below 0.01. This result revealed the low genetic diversity across the entire mitogenome of *Z. latifolia*. Comparatively, wild rice exhibits the highest nucleotide diversity, followed by landrace, while weedy rice displays the lowest diversity (Tong et al., 2017). These genes with high nucleotide diversity play a pivotal role in exploring the origin and evolution of germplasm resources within the *Zizania* mitogenome. This is an important contribution to expanding the genetic base, identifying beneficial genes in germplasm resources from different genetic backgrounds, and understanding differences between individuals.

### 4.3 Phylogenetic and evolutionary analyses of *Z. latifolia*

The Poaceae family comprises approximately 1,000 species, encompassing vital cereal crops such as wheat, rice, and maize. Phylogenetic analyses of the Poaceae family, based on 30 published complete mitogenome sequences, indicated that *Z. latifolia* was sister to the Oryzeae clade within the subfamily Oryzoideae. Notably, Oryzoideae (including *Z. latifolia*) was only a sister to Pooideae. This finding was consistent with the topological structure derived from chloroplast genome-based phylogenetic analyses (Zhang et al., 2016). Previous studies utilizing nuclear and plastid data consistently identified two major branches within the Poaceae family—BOP (Bambusoideae, Oryzoideae, and Pooideae) and PACMAD (Panicoideae, Arundinoideae, Chloridoideae, Micrairoideae, Aristidoideae, and Danthonioideae)—corroborating our research conclusions (Burke et al., 2016; Huang et al., 2022). Our results contributed to the understanding of mitogenomic information in Poaceae, enabling a detailed description of evolutionary relationships and more effective interspecific molecular breeding (Choi et al., 2019; Yang et al., 2021; Xia et al., 2022).

Collinearity analysis revealed numerous homologous regions among the eight Poaceae species. The varying relative positions of these homologous regions indicated abundant rearrangements and inversions within the eight mitogenomes. Notably, greater synteny was observed between *Z. latifolia* and *O. coarctata*, indicating a higher similarity in sequence composition and arrangement, consistent with the phylogenetic tree results.

Ka/Ks analysis of the mitogenomes of *Z. latifolia*, *O. coarctata*, *O. sativa*, *T. aestivum*, and *Z. perennis* showed that the Ka/Ks values of most PCGs were less than 1, consistent with previous studies (Cheng et al., 2021; Ma et al., 2022). However, our study identified genes with Ka/Ks > 1, such as *sdh3*, suggesting significant roles in evolutionary history and essential life activities.

### 4.4 Comparison genome analysis

Plant mitogenomes exhibit variability in gene content and structure (Kumar et al., 2016). Here, we conducted a comparative analysis of mitogenome genes across 30 species to elucidate gene duplication and loss events (Atluri et al., 2015; Hall et al., 2020). The results indicated that gene losses, including *rpl14*, *rpl33*, *sdh3*, *rps10*, and *rps11*, predated the formation of the Poaceae family. Within the Poaceae family, the *sdh4* gene was exclusive to *A. longiglumis*, *rpl5* was entirely lost in Chloridoideae, *mttB* was absent in the Oryzoideae subfamily, *rps19* was completely lost in Panicoideae, and *rps14* was only found in *Lolium perenne* and *A. longiglumis*. Notably, all other species within the family had lost the *rps14* gene. In the *Z. latifolia* mitogenome, *cox2*, *rrn5*, *rrn18*, and *rrn26* all had two copies, which was similar to the genus *Saccharum* within Poaceae. Multiple copies of genes influence gene expression in plant mitochondria, which may lead to enhanced function t in adapting to different environments. These genes may affect the translocation and splicing of mitochondrial genes (Kwasniak-Owczarek et al., 2019), which further affects the development, reproduction, and other morphological and physiological traits of plants (Zhang et al., 2006).

In addition, we compared the size and GC content in the mitogenomes of 30 species. Most species showed only small differences in genome size, except for *A. trichopoda*, which exceeded 3 Mb due to the absorption of sequences from plastid or nuclear genome (Rice et al., 2013). *G. biloba* had a high GC content of 50%, while other species had smaller differences in GC content. This indicated a stable evolution of mitogenomes over time, supporting that the GC content remained consistent (Cheng et al., 2021).

## 5 Conclusions

Here, we assembled and annotated the mitogenome of *Z. latifolia*, and comparative genomic analyses were performed. The mitogenome of *Z. latifolia* was 392,219 bp, containing 33 PCGs, 13 tRNA genes, and three rRNA genes. There were 46 SSRs, and eight high-nucleotide polymorphic regions were identified in the mitogenome of *Z. latifolia*, which were potential loci for the development of molecular markers for phylogenetic analysis and species identification of *Z. latifolia*. Synteny analysis and phylogenetic analysis revealed that *Z. latifolia* was most closely related to *O. coarctata* of the genus *Oryza*, which provided a kinship basis for wild rice germplasm introgression. This study provided valuable insights into the mitochondrial genomic resources of *Z. latifolia* and contributed an important theoretical foundation for the conservation of the germplasm diversity and the yield improvement of *Z. latifolia*.

## Data Availability

The original contributions presented in the study are publicly available. This data can be found at the National Center for Biotechnology Information (NCBI) using accession number PRJNA1064687.
