# Supplementary material for: Complete mitochondrial genome assembly of Zizania latifolia and comparative genome analysis
Source: Front Plant Sci. 2024 Aug 9;15:1381089. doi: 10.3389/fpls.2024.1381089 (PMC11341417; doi:10.3389/fpls.2024.1381089)
Supplement: Supplementary file 1 [file DataSheet_1.docx]

***Supplementary Material***

Complete mitochondrial genome assembly of *Zizania latifolia* and comparative genome analysis

**Xianyang Luo^1^, Cuicui Gu^1^, Sijia Gao^1^, Man Li^1^, Haixiang Zhang^1^, Shidong Zhu^1*^**

^1^ College of Horticulture, Anhui Agricultural University, Hefei 230036, China

*** Correspondence:**Shidong Zhu
sdzhuaau@sina.cn

**1 Supplementary Tables**

| Next-generation sequencing platform | | | | | | Third-generation sequencing platform | | | |
| --- | --- | --- | --- | --- | --- | --- | --- | --- | --- |
| Clean bases | Clean reads (bp) | Read length (bp) | Q20 (%) | Q30 (%) | GC (%) | Number of Reads | Number of Bases | Mean length (bp) | N50 length (bp) |
| 309568178 | 92870453400 | 150 | 95.76 | 89.33 | 41.87 | 3626579 | 50156272680 | 13830 | 15210 |

**Supplementary Table 1.** Summary of Sequencing statistics.

**Supplementary Table 2.** Distribution of SSRs in *Z. latifolia* mitochondrial and chloroplast genome.

|  | Mitogenome | | | | Chloroplast | | | |
| --- | --- | --- | --- | --- | --- | --- | --- | --- |
| SSR type | SSR | size | start | end | SSR | size | start | end |
| p1 | (T)10 | 10 | 15591 | 15600 | (A)10 | 10 | 11872 | 11881 |
|  | (A)10 | 10 | 29514 | 29523 | (A)12 | 12 | 12258 | 12269 |
|  | (T)11 | 11 | 32266 | 32276 | (C)11 | 11 | 13959 | 13969 |
|  | (A)10 | 10 | 63093 | 63102 | (A)11 | 11 | 30254 | 30264 |
|  | (T)10 | 10 | 89526 | 89535 | (T)10 | 10 | 42544 | 42553 |
|  | (T)10 | 10 | 89692 | 89701 | (A)15 | 15 | 44857 | 44871 |
|  | (T)10 | 10 | 92731 | 92740 | (A)10 | 10 | 47184 | 47193 |
|  | (T)10 | 10 | 138667 | 138676 | (T)12 | 12 | 50306 | 50317 |
|  | (T)10 | 10 | 151904 | 151913 | (T)10 | 10 | 55195 | 55204 |
|  | (A)10 | 10 | 152265 | 152274 | (A)10 | 10 | 73039 | 73048 |
|  | (C)10 | 10 | 166786 | 166795 | (T)10 | 10 | 73552 | 73561 |
|  | (A)10 | 10 | 188551 | 188560 | (G)15 | 15 | 79270 | 79284 |
|  | (A)10 | 10 | 199540 | 199549 | (T)10 | 10 | 81642 | 81651 |
|  | (A)13 | 13 | 248391 | 248403 | - | - | - | - |
|  | (T)11 | 11 | 249063 | 249073 | - | - | - | - |
|  | (A)12 | 12 | 255868 | 255879 | - | - | - | - |
|  | (T)11 | 11 | 285418 | 285428 | - | - | - | - |
|  | (G)11 | 11 | 293612 | 293622 | - | - | - | - |
|  | (A)10 | 10 | 348704 | 348713 | - | - | - | - |
|  | (A)10 | 10 | 365442 | 365451 | - | - | - | - |
| p2 | (AT)6 | 12 | 4509 | 4520 | (AT)5 | 10 | 26615 | 26624 |
|  | (CT)5 | 10 | 19274 | 19283 | (TC)5 | 10 | 45226 | 45235 |
|  | (TA)5 | 10 | 51044 | 51053 | (TA)5 | 10 | 66120 | 66129 |
|  | (TC)5 | 10 | 59015 | 59024 | - | - | - | - |
|  | (TA)6 | 12 | 103087 | 103098 | - | - | - | - |
|  | (AT)5 | 10 | 104839 | 104848 | - | - | - | - |
|  | (AG)5 | 10 | 123355 | 123364 | - | - | - | - |
|  | (CT)5 | 10 | 126953 | 126962 | - | - | - | - |
|  | (TC)5 | 10 | 150774 | 150783 | - | - | - | - |
|  | (TA)11 | 22 | 152072 | 152093 | - | - | - | - |
|  | (AG)5 | 10 | 173928 | 173937 | - | - | - | - |
|  | (CT)5 | 10 | 186342 | 186351 | - | - | - | - |
|  | (TA)5 | 10 | 200905 | 200914 | - | - | - | - |
|  | (CT)5 | 10 | 221643 | 221652 | - | - | - | - |
|  | (TA)6 | 12 | 314515 | 314526 | - | - | - | - |
|  | (GA)5 | 10 | 337715 | 337724 | - | - | - | - |
|  | (TA)5 | 10 | 339322 | 339331 | - | - | - | - |
|  | (CT)5 | 10 | 348260 | 348269 | - | - | - | - |
|  | (GA)5 | 10 | 377155 | 377164 | - | - | - | - |
|  | (TC)5 | 10 | 380753 | 380762 | - | - | - | - |
| p4 | (CTAC)4 | 16 | 191247 | 191262 | (GTAG)4 | 16 | 52493 | 52508 |
| p5 | (CGGGC)3 | 15 | 67164 | 67178 | (ATAAA)3 | 15 | 3942 | 3956 |
|  | (GTATG)3 | 15 | 211340 | 211354 | - | - | - | - |
|  | (ATCAT)3 | 15 | 324110 | 324124 | - | - | - | - |
|  | (AAAGA)3 | 15 | 334493 | 334507 | - | - | - | - |
| p6 | - | - | - | - | (TTTCTA)3 | 18 | 17954 | 17971 |
| c | (CT)7attttctttgatgcaatttaga  gagaaagagcagtgcgaactag(A)10 | 68 | 66814 | 66881 | (T)10ctctccta(T)10ctgtcata(T)10 | 46 | 78129 | 78174 |

**Supplementary Table 3.** Distribution of perfect tandem repeats in *Z. latifolia* mitogenome.

| NO. | Size | Repeat sequence | Copy | Percent Matches | Start | End |
| --- | --- | --- | --- | --- | --- | --- |
| 1 | 84 | TTCGACCCAGCCCCAAATATTGATCTTGAGTTGCACATGGATCCGCCTGAACCGCAACAGGGGGCCCCCACCCCCCAACAAGTA | 2.1 | 93 | 967 | 1142 |
| 2 | 16 | CTTTACTACGATATCT | 2.2 | 95 | 11838 | 11873 |
| 3 | 33 | TCCCGTATTCGTCCTCTGAGTCTTCCTCTAAAA | 2.3 | 95 | 34380 | 34454 |
| 4 | 21 | ATATTCGTCCTCTAGGATCCC | 6.6 | 71 | 34525 | 34663 |
| 5 | 54 | TCCTATATTCGTCCTCTAGGATCCCATATTCGTCCTCTGAGTCTTCCTCTCGAG | 5.3 | 72 | 34467 | 34749 |
| 6 | 42 | ATATTCGTCCTCTAGGATCCCATATTCATCTTCTAGGGTTTC | 2.4 | 94 | 34525 | 34623 |
| 7 | 14 | CTTAGATGAAATAG | 2 | 100 | 59257 | 59284 |
| 8 | 21 | ATTTTCATGGTAGGAAAGGAT | 2.2 | 92 | 72503 | 72548 |
| 9 | 51 | CTAACTCGGCCCGCGAGTCACAAGTTTTTGGTAGAATGGGGGTAGGGGGG | 3 | 90 | 72946 | 73097 |
| 10 | 19 | TGAACTACTTGAGCTGCCCCCCCT | 3.1 | 60 | 73125 | 73192 |
| 11 | 42 | GGGCTGACGTCCCCTGAACTACTTGAGCTGCCCCCCCTTGAA | 2 | 97 | 73111 | 73192 |
| 12 | 28 | TACTTATTATAGCAAGCATCCTCTTAGC | 2 | 96 | 100350 | 100405 |
| 13 | 25 | TATGTCAACCTACCCCTCCTATCGT | 2 | 88 | 102336 | 102385 |
| 14 | 27 | TAGAAAGGCAGAGACACCTATGACCAG | 2 | 100 | 108937 | 108990 |
| 15 | 11 | GAAAGAAATAA | 2.5 | 100 | 167533 | 167560 |
| 16 | 26 | ATGTGTGCTTTATGACCAATAAATAA | 2 | 100 | 183247 | 183298 |
| 17 | 13 | CGAATCCGTATAG | 2.1 | 100 | 192161 | 192187 |
| 18 | 33 | AGTAAACTAGTGGCTTCTTCTTCTTGTGCTTCT | 1.9 | 100 | 193138 | 193201 |
| 19 | 18 | ATCTTTACTTAATATAGA | 2 | 100 | 225644 | 225679 |
| 20 | 27 | GAGGAGCCAATATACATCCTAGTTCAT | 2 | 96 | 231773 | 231826 |
| 21 | 26 | TTCGGAAATGTCTAACAAGGGACTCT | 2.1 | 100 | 231826 | 231879 |
| 22 | 23 | TCTTAGTACTACCGCTTTCCATT | 2 | 95 | 257309 | 257354 |
| 23 | 12 | TCTCTGGTGCCA | 2.2 | 100 | 264107 | 264132 |
| 24 | 41 | ATCAAACTAAACAAAGACACTTAGCATAGGAGTAGATCTCC | 2.4 | 96 | 283567 | 283666 |
| 25 | 13 | ATTGATGACTAAT | 2.1 | 100 | 287396 | 287422 |
| 26 | 22 | ATTGGCAAAATTCGGTGCTATC | 2.1 | 100 | 292274 | 292319 |
| 27 | 34 | AACGAATAAAACCCCTTGTCGAAGCCCTTTATGA | 3 | 98 | 303249 | 303350 |
| 28 | 16 | AATACCTGACTATTTA | 3 | 84 | 316531 | 316577 |
| 29 | 28 | ATCTATTCTTTCATGACGTGAATATGTG | 3 | 68 | 318385 | 318468 |
| 30 | 64 | CGCTGCTCGTGGTGGAGTACTCTCTGAATTATTCGCTCTATTATTGCCTCAGGATGTGATCGGG | 2 | 100 | 318591 | 318718 |
| 31 | 24 | AGATACAATGAAAGTCAGTCTTCA | 1.9 | 95 | 321155 | 321200 |
| 32 | 39 | AAATAAAGCCTTGCACCTCCATGTGACCTAACAGGACTC | 2 | 94 | 326377 | 326453 |
| 33 | 17 | ATATATTATATATATATT | 2 | 88 | 328205 | 328239 |
| 34 | 22 | TGTCAATCTCTGTTGCAGGAGT | 3.2 | 78 | 341161 | 341231 |
| 35 | 19 | GACGAGGACTAGTGAGGCT | 2 | 100 | 348511 | 348548 |
| 36 | 19 | TATTGAATCTGGCTTCTAT | 2.5 | 89 | 351671 | 351718 |

**Supplementary Table 4.** Relative synonymous codon RSCU usage values of the *Z. latifolia* mitogenome.

| Codon | AA | No. | RSCU | Codon | AA | No. | RSCU |
| --- | --- | --- | --- | --- | --- | --- | --- |
| UUU | Phe | 380 | 1.16 | GCA | Ala | 146 | 0.92 |
| UUC | Phe | 275 | 0.84 | GCG | Ala | 93 | 0.59 |
| UUA | Leu2 | 258 | 1.42 | UAU | Tyr | 231 | 1.43 |
| UUG | Leu2 | 209 | 1.15 | UAC | Tyr | 92 | 0.57 |
| CUU | Leu1 | 244 | 1.34 | CAU | His | 221 | 1.51 |
| CUC | Leu1 | 124 | 0.68 | CAC | His | 72 | 0.49 |
| CUA | Leu1 | 141 | 0.77 | CAA | Gln | 233 | 1.51 |
| CUG | Leu1 | 117 | 0.64 | CAG | Gln | 75 | 0.49 |
| AUU | Ile | 342 | 1.3 | AAU | Asn | 242 | 1.35 |
| AUC | Ile | 222 | 0.84 | AAC | Asn | 117 | 0.65 |
| AUA | Ile | 226 | 0.86 | AAA | Lys | 275 | 1.19 |
| AUG | Met | 249 | 1 | AAG | Lys | 189 | 0.81 |
| GUU | Val | 203 | 1.24 | GAU | Asp | 245 | 1.36 |
| GUC | Val | 126 | 0.77 | GAC | Asp | 114 | 0.64 |
| GUA | Val | 179 | 1.09 | GAA | Glu | 282 | 1.39 |
| GUG | Val | 147 | 0.9 | GAG | Glu | 125 | 0.61 |
| UCU | Ser2 | 198 | 1.28 | UGU | Cys | 93 | 1.06 |
| UCC | Ser2 | 166 | 1.07 | UGC | Cys | 83 | 0.94 |
| UCA | Ser2 | 167 | 1.08 | UGG | Trp | 157 | 1 |
| UCG | Ser2 | 125 | 0.81 | CGU | Arg | 140 | 1.14 |
| CCU | Pro | 188 | 1.36 | CGC | Arg | 76 | 0.62 |
| CCC | Pro | 124 | 0.9 | CGA | Arg | 155 | 1.26 |
| CCA | Pro | 168 | 1.21 | CGG | Arg | 99 | 0.8 |
| CCG | Pro | 74 | 0.53 | AGU | Ser1 | 155 | 1 |
| ACU | Thr | 189 | 1.47 | AGC | Ser1 | 119 | 0.77 |
| ACC | Thr | 127 | 0.98 | AGA | Arg | 174 | 1.41 |
| ACA | Thr | 126 | 0.98 | AGG | Arg | 96 | 0.78 |
| ACG | Thr | 74 | 0.57 | GGU | Gly | 212 | 1.15 |
| GCU | Ala | 252 | 1.59 | GGC | Gly | 107 | 0.58 |
| GCC | Ala | 141 | 0.89 | GGA | Gly | 257 | 1.4 |
|  |  |  |  | GGG | Gly | 160 | 0.87 |

**Supplementary Table 5.** Distribution of RNA editing in *Z. latifolia* mitogenome.

| #Chr | Strand | ***Name*** | Genome_pos | Gene_pos | AA_pos | Phase | Ref->Alt | RefCodon->AltCodon | RefAA->AltAA | AltRatio |
| --- | --- | --- | --- | --- | --- | --- | --- | --- | --- | --- |
| *Zizania* | + | *atp1* | 180971 | 1490 | 497 | 2 | C->T | CCT->CTT | P->L | 1 |
| *Zizania* | + | *atp1* | 179511 | 30 | 10 | 3 | C->T | CTC->CTT | L->L | 0.8 |
| *Zizania* | + | *atp1* | 180659 | 1178 | 393 | 2 | C->T | TCA->TTA | S->L | 0.714286 |
| *Zizania* | + | *atp1* | 180980 | 1499 | 500 | 2 | C->T | TCT->TTT | S->F | 0.933333 |
| *Zizania* | - | *atp4* | 293115 | 414 | 138 | 3 | C->T | CAC->CAT | H->H | 1 |
| *Zizania* | - | *atp4* | 293136 | 393 | 131 | 3 | C->T | GTC->GTT | V->V | 0.5 |
| *Zizania* | + | *atp6* | 350191 | 502 | 168 | 1 | C->T | CAT->TAT | H->Y | 0.888889 |
| *Zizania* | + | *atp6* | 349765 | 76 | 26 | 1 | C->T | CCA->TCA | P->S | 1 |
| *Zizania* | + | *atp6* | 349957 | 268 | 90 | 1 | C->T | CGC->TGC | R->C | 1 |
| *Zizania* | + | *atp6* | 349990 | 301 | 101 | 1 | C->T | CGT->TGT | R->C | 1 |
| *Zizania* | + | *atp6* | 350255 | 566 | 189 | 2 | C->T | TCA->TTA | S->L | 0.833333 |
| *Zizania* | + | *atp6* | 350213 | 524 | 175 | 2 | C->T | TCA->TTA | S->L | 0.888889 |
| *Zizania* | + | *atp6* | 350384 | 695 | 232 | 2 | C->T | TCA->TTA | S->L | 1 |
| *Zizania* | + | *atp6* | 350408 | 719 | 240 | 2 | C->T | TCA->TTA | S->L | 1 |
| *Zizania* | + | *atp6* | 349982 | 293 | 98 | 2 | C->T | TCG->TTG | S->L | 0.818182 |
| *Zizania* | + | *atp6* | 349964 | 275 | 92 | 2 | C->T | TCG->TTG | S->L | 1 |
| *Zizania* | + | *atp6* | 349775 | 86 | 29 | 2 | C->T | TCT->TTT | S->F | 1 |
| *Zizania* | - | *atp8* | 112018 | 200 | 67 | 2 | C->T | TCG->TTG | S->L | 1 |
| *Zizania* | - | *atp9* | 338308 | 191 | 64 | 2 | C->T | CCA->CTA | P->L | 1 |
| *Zizania* | - | *atp9* | 338276 | 223 | 75 | 1 | C->T | CGA->TGA | R->* | 0.866667 |
| *Zizania* | - | *atp9* | 338417 | 82 | 28 | 1 | C->T | CTC->TTC | L->F | 0.885714 |
| *Zizania* | - | *atp9* | 338365 | 134 | 45 | 2 | C->T | TCA->TTA | S->L | 0.902439 |
| *Zizania* | - | *atp9* | 338479 | 20 | 7 | 2 | C->T | TCA->TTA | S->L | 1 |
| *Zizania* | - | *atp9* | 338287 | 212 | 71 | 2 | C->T | TCA->TTA | S->L | 1 |
| *Zizania* | - | *atp9* | 338407 | 92 | 31 | 2 | C->T | TCG->TTG | S->L | 0.833333 |
| *Zizania* | + | *ccmC* | 199403 | 677 | 226 | 2 | T->C | TTA->TCA | L->S | 0.83871 |
| *Zizania* | + | *cox1* | 326780 | 185 | 62 | 2 | C->T | ACG->ATG | T->M | 0.6 |
| *Zizania* | + | *cox1* | 326984 | 389 | 130 | 2 | C->T | ACG->ATG | T->M | 0.941176 |
| *Zizania* | + | *cox1* | 326787 | 192 | 64 | 3 | C->T | CAC->CAT | H->H | 1 |
| *Zizania* | + | *cox1* | 328084 | 1489 | 497 | 1 | C->T | CCA->TCA | P->S | 1 |
| *Zizania* | + | *cox1* | 326730 | 135 | 45 | 3 | C->T | CCC->CCT | P->P | 0.608696 |
| *Zizania* | + | *cox1* | 326993 | 398 | 133 | 2 | C->T | CCG->CTG | P->L | 0.888889 |
| *Zizania* | + | *cox1* | 326725 | 130 | 44 | 1 | C->T | CGA->TGA | R->* | 0.583333 |
| *Zizania* | + | *cox1* | 326938 | 343 | 115 | 1 | C->A | CTA->ATA | L->I | 1 |
| *Zizania* | + | *cox1* | 326929 | 334 | 112 | 1 | C->T | CTC->TTC | L->F | 1 |
| *Zizania* | + | *cox1* | 326744 | 149 | 50 | 2 | T->C | CTT->CCT | L->P | 0.55 |
| *Zizania* | + | *cox1* | 327026 | 431 | 144 | 2 | G->A | GGA->GAA | G->E | 0.8 |
| *Zizania* | + | *cox1* | 326610 | 15 | 5 | 3 | C->T | GTC->GTT | V->V | 1 |
| *Zizania* | + | *cox1* | 328028 | 1433 | 478 | 2 | C->T | TCA->TTA | S->L | 1 |
| *Zizania* | + | *cox1* | 326954 | 359 | 120 | 2 | T->A | TTA->TAA | L->* | 0.941176 |
| *Zizania* | + | *cox3* | 14926 | 458 | 153 | 2 | C->T | CCT->CTT | P->L | 1 |
| *Zizania* | + | *cox3* | 14935 | 467 | 156 | 2 | C->T | CCT->CTT | P->L | 1 |
| *Zizania* | + | *cox3* | 15079 | 611 | 204 | 2 | C->T | TCC->TTC | S->F | 1 |
| *Zizania* | + | *cox3* | 14827 | 359 | 120 | 2 | C->T | TCT->TTT | S->F | 0.875 |
| *Zizania* | + | *mat-r* | 253818 | 1706 | 569 | 2 | C->T | CCT->CTT | P->L | 1 |
| *Zizania* | + | *mat-r* | 253797 | 1685 | 562 | 2 | C->T | TCC->TTC | S->F | 0.75 |
| *Zizania* | + | *nad3* | 296231 | 79 | 27 | 1 | C->T | CAT->TAT | H->Y | 1 |
| *Zizania* | + | *nad3* | 296288 | 136 | 46 | 1 | C->T | CCG->TCG | P->S | 1 |
| *Zizania* | + | *nad3* | 296297 | 145 | 49 | 1 | C->T | CCG->TCG | P->S | 1 |
| *Zizania* | + | *nad3* | 296195 | 43 | 15 | 1 | C->T | CGC->TGC | R->C | 1 |
| *Zizania* | + | *nad3* | 296366 | 214 | 72 | 1 | C->T | CGG->TGG | R->W | 0.8 |
| *Zizania* | + | *nad3* | 296495 | 343 | 115 | 1 | C->T | CGG->TGG | R->W | 0.818182 |
| *Zizania* | + | *nad3* | 296336 | 184 | 62 | 1 | C->T | CGG->TGG | R->W | 1 |
| *Zizania* | + | *nad3* | 296402 | 250 | 84 | 1 | C->T | CTA->TTA | L->L | 0.75 |
| *Zizania* | + | *nad3* | 296468 | 316 | 106 | 1 | C->T | CTC->TTC | L->F | 1 |
| *Zizania* | + | *nad3* | 296381 | 229 | 77 | 1 | C->T | CTT->TTT | L->F | 0.833333 |
| *Zizania* | + | *nad3* | 296426 | 274 | 92 | 1 | C->T | CTT->TTT | L->F | 0.909091 |
| *Zizania* | + | *nad3* | 296398 | 246 | 82 | 3 | C->T | TAC->TAT | Y->Y | 1 |
| *Zizania* | + | *nad3* | 296341 | 189 | 63 | 3 | C->T | TTC->TTT | F->F | 1 |
| *Zizania* | - | *nad4* | 46188 | 44 | 15 | 2 | C->T | CCT->CTT | P->L | 1 |
| *Zizania* | - | *nad4* | 46155 | 77 | 26 | 2 | C->T | CCT->CTT | P->L | 1 |
| *Zizania* | + | *nad6* | 200165 | 446 | 149 | 2 | C->T | TCC->TTC | S->F | 0.9375 |
| *Zizania* | + | *nad6* | 200288 | 569 | 190 | 2 | C->T | TCT->TTT | S->F | 1 |
| *Zizania* | - | *nad7* | 161622 | 1088 | 363 | 2 | C->T | TCA->TTA | S->L | 1 |
| *Zizania* | + | *nad9* | 63483 | 113 | 38 | 2 | C->T | CCA->CTA | P->L | 1 |
| *Zizania* | + | *nad9* | 63462 | 92 | 31 | 2 | C->T | TCT->TTT | S->F | 1 |
| *Zizania* | - | *rpl16* | 312877 | 457 | 153 | 1 | C->T | CAT->TAT | H->Y | 1 |
| *Zizania* | - | *rpl16* | 312811 | 523 | 175 | 1 | C->T | CAT->TAT | H->Y | 1 |
| *Zizania* | - | *rpl16* | 312891 | 443 | 148 | 2 | C->T | CCA->CTA | P->L | 0.833333 |
| *Zizania* | - | *rpl16* | 312805 | 529 | 177 | 1 | C->T | CGT->TGT | R->C | 1 |
| *Zizania* | - | *rpl16* | 313242 | 92 | 31 | 2 | C->T | GCG->GTG | A->V | 0.666667 |
| *Zizania* | - | *rpl16* | 313287 | 47 | 16 | 2 | C->T | TCT->TTT | S->F | 0.684211 |
| *Zizania* | - | *rpl16* | 313256 | 78 | 26 | 3 | C->T | TTC->TTT | F->F | 0.777778 |
| *Zizania* | + | *rpl2* | 178832 | 1362 | 454 | 3 | T->C | ATT->ATC | I->I | 0.6875 |
| *Zizania* | + | *rpl2* | 178841 | 1371 | 457 | 3 | A->G | CCA->CCG | P->P | 0.722222 |
| *Zizania* | + | *rpl2* | 178902 | 1432 | 478 | 1 | C->T | CGA->TGA | R->* | 0.631579 |
| *Zizania* | + | *rpl2* | 178896 | 1426 | 476 | 1 | C->T | CGA->TGA | R->* | 0.666667 |
| *Zizania* | + | *rpl2* | 178593 | 1123 | 375 | 1 | C->T | CGG->TGG | R->W | 1 |
| *Zizania* | + | *rpl2* | 178804 | 1334 | 445 | 2 | G->A | CGT->CAT | R->H | 0.722222 |
| *Zizania* | + | *rpl2* | 178855 | 1385 | 462 | 2 | T->C | CTT->CCT | L->P | 0.73913 |
| *Zizania* | + | *rpl2* | 178619 | 1149 | 383 | 3 | G->A | GCG->GCA | A->A | 1 |
| *Zizania* | + | *rpl2* | 178507 | 1037 | 346 | 2 | C->T | GCG->GTG | A->V | 0.9375 |
| *Zizania* | + | *rpl2* | 178708 | 1238 | 413 | 2 | C->T | TCA->TTA | S->L | 0.928571 |
| *Zizania* | + | *rps12* | 296754 | 196 | 66 | 1 | C->T | CAC->TAC | H->Y | 1 |
| *Zizania* | + | *rps12* | 296847 | 289 | 97 | 1 | C->T | CGT->TGT | R->C | 1 |
| *Zizania* | + | *rps12* | 296842 | 284 | 95 | 2 | C->T | TCC->TTC | S->F | 1 |
| *Zizania* | + | *rps12* | 296827 | 269 | 90 | 2 | C->T | TCG->TTG | S->L | 0.857143 |
| *Zizania* | + | *rps12* | 296629 | 71 | 24 | 2 | C->T | TCG->TTG | S->L | 1 |
| *Zizania* | + | *rps12* | 296779 | 221 | 74 | 2 | C->T | TCG->TTG | S->L | 1 |
| *Zizania* | - | *rps13* | 29889 | 100 | 34 | 1 | C->T | CGT->TGT | R->C | 0.833333 |
| *Zizania* | + | *rps19* | 347735 | 164 | 55 | 2 | C->T | CCT->CTT | P->L | 0.833333 |
| *Zizania* | - | *rps4* | 311560 | 10 | 4 | 1 | T->A | TTA->ATA | L->I | 0.875 |
| *Zizania* | - | *trnN-GUU* | 263192 | 24 | ? | ? | C->T | ?->? | ?->? | 0.636364 |

**Supplementary Table 6.** Prediction of RNA editing sites.

| Type | RNA -editing | Number | Percentage |
| --- | --- | --- | --- |
| hydrophobic | CTA(L)->ATA(I) | 1 | 34.8% |
|  | CTA(L)->TTA(L) | 1 |  |
|  | CTC(L)->CTT(L) | 1 |  |
|  | CTC(L)->TTC(F) | 3 |  |
|  | CTT(L)->CCT(P) | 2 |  |
|  | CTT(L)->TTT(F) | 2 |  |
|  | GCG(A)->GCA(A) | 1 |  |
|  | GCG(A)->GTG(V) | 2 |  |
|  | CCT(P)->CTT(L) | 7 |  |
|  | GTC(V)->GTT(V) | 2 |  |
|  | ATT(I)->ATC(I) | 1 |  |
|  | CCA(P)->CCG(P) | 1 |  |
|  | CCA(P)->CTA(L) | 3 |  |
|  | CCC(P)->CCT(P) | 1 |  |
|  | CCG(P)->CTG(L) | 1 |  |
|  | TTA(L)->ATA(I) | 1 |  |
|  | TTC(F)->TTT(F) | 2 |  |
| hydrophilic | CAC(H)->CAT(H) | 2 | 16.3% |
|  | CAC(H)->TAC(Y) | 1 |  |
|  | CAT(H)->TAT(Y) | 4 |  |
|  | CGT(R)->CAT(H) | 1 |  |
|  | CGT(R)->TGT(C) | 4 |  |
|  | TAC(Y)->TAT(Y) | 1 |  |
|  | CGC(R)->TGC(C) | 2 |  |
| hydrophilic-hydrophobic | TCA(S)->TTA(L) | 11 | 36.9% |
|  | TCC(S)->TTC(F) | 4 |  |
|  | TCG(S)->TTG(L) | 7 |  |
|  | TCT(S)->TTT(F) | 6 |  |
|  | CGG(R)->TGG(W) | 4 |  |
|  | ACG(T)->ATG(M) | 2 |  |
| hydrophobic-hydrophilic | TTA(L)->TCA(S) | 1 | 6.5% |
|  | GGA(G)->GAA(E) | 1 |  |
|  | CCA(P)->TCA(S) | 2 |  |
|  | CCG(P)->TCG(S) | 2 |  |
| hydrophobic-stop | TTA(L)->TAA (*) | 1 | 1.08% |
| hydrophilic-stop | CGA(R)->TGA (*) | 4 | 4.35% |

**Supplementary Table 7.** Chloroplast and mitochondrial genomes share a homologous region.

|  | Length  (bp) | Identity  (%) | Mis match | Gap opens | Cp start  (bp) | Cp end  (bp) | Mt start (bp) | Mt end (bp) | Gene annotation |
| --- | --- | --- | --- | --- | --- | --- | --- | --- | --- |
| mtpt1 | 6226 | 99.936 | 4 | 0 | 130148 | 136373 | 305972 | 299747 | / |
| mtpt2 | 6226 | 99.936 | 4 | 0 | 82288 | 88513 | 299747 | 305972 | *trnH-GUG* |
| mtpt3 | 5666 | 99.965 | 1 | 1 | 26616 | 32280 | 35915 | 30250 | / |
| mtpt4 | 5327 | 98.63 | 43 | 16 | 51378 | 56700 | 192355 | 187055 | *trnW-CAU* |
| mtpt5 | 3571 | 99.972 | 1 | 0 | 96290 | 99860 | 292714 | 289144 | / |
| mtpt6 | 3571 | 99.972 | 1 | 0 | 118801 | 122371 | 289144 | 292714 | / |
| mtpt7 | 1904 | 99.055 | 16 | 2 | 48361 | 50263 | 61015 | 62917 | *trnF-GAA* |
| mtpt8 | 1639 | 99.939 | 1 | 0 | 123150 | 124788 | 289144 | 287506 | / |
| mtpt9 | 1639 | 99.939 | 1 | 0 | 93873 | 95511 | 287506 | 289144 | / |
| mtpt10 | 1289 | 99.69 | 3 | 1 | 70099 | 71386 | 35899 | 37187 | / |
| mtpt11 | 739 | 99.729 | 1 | 1 | 112641 | 113379 | 293685 | 294422 | / |
| mtpt12 | 1098 | 87.705 | 109 | 21 | 45492 | 46579 | 59333 | 60414 | *trnA-GGA* |
| mtpt13 | 644 | 92.547 | 29 | 9 | 111779 | 112403 | 339396 | 340039 | / |
| mtpt14 | 287 | 97.909 | 6 | 0 | 57003 | 57289 | 301984 | 301698 | / |
| mtpt15 | 343 | 90.962 | 29 | 2 | 45136 | 45476 | 58925 | 59267 | / |
| mtpt16 | 374 | 89.305 | 27 | 7 | 113011 | 113382 | 239270 | 238908 | / |
| mtpt17 | 886 | 74.153 | 180 | 38 | 124692 | 125555 | 136605 | 135747 | / |
| mtpt18 | 886 | 74.153 | 180 | 38 | 93106 | 93969 | 135747 | 136605 | / |
| mtpt19 | 886 | 74.153 | 180 | 38 | 93106 | 93969 | 368371 | 367513 | *rrn18*(54.2%) |
| mtpt20 | 886 | 74.153 | 180 | 38 | 124692 | 125555 | 367513 | 368371 | *rrn18*(54.2%) |
| mtpt21 | 238 | 85.714 | 17 | 10 | 65794 | 66030 | 151466 | 151687 | / |
| mtpt22 | 404 | 77.723 | 61 | 18 | 47851 | 48229 | 60514 | 60913 | / |
| mtpt23 | 119 | 100 | 0 | 0 | 92705 | 92823 | 86235 | 86117 | / |
| mtpt24 | 119 | 100 | 0 | 0 | 125838 | 125956 | 86117 | 86235 | / |
| mtpt25 | 258 | 81.395 | 32 | 6 | 19040 | 19288 | 13370 | 13120 | *trnC-GCA* |
| mtpt26 | 184 | 85.326 | 11 | 8 | 122564 | 122745 | 99639 | 99470 | / |
| mtpt27 | 184 | 85.326 | 11 | 8 | 95916 | 96097 | 99470 | 99639 | / |
| mtpt28 | 86 | 100 | 0 | 0 | 117749 | 117834 | 263216 | 263131 | *trnW-GUU* |
| mtpt29 | 86 | 100 | 0 | 0 | 100827 | 100912 | 263131 | 263216 | *trnW-GUU* |
| mtpt30 | 114 | 92.105 | 5 | 1 | 96926 | 97039 | 47386 | 47277 | / |
| mtpt31 | 114 | 92.105 | 5 | 1 | 121622 | 121735 | 47277 | 47386 | / |
| mtpt32 | 124 | 88.71 | 12 | 2 | 65548 | 65670 | 151209 | 151331 | *trnW-CCA* |
| mtpt33 | 197 | 81.218 | 23 | 9 | 27768 | 27963 | 34708 | 34525 | / |
| mtpt34 | 197 | 81.218 | 23 | 9 | 27822 | 28005 | 34762 | 34567 | / |
| mtpt35 | 68 | 98.529 | 1 | 0 | 132770 | 132837 | 303316 | 303249 | / |
| mtpt36 | 68 | 98.529 | 1 | 0 | 85824 | 85891 | 303249 | 303316 | / |
| mtpt37 | 68 | 98.529 | 1 | 0 | 132804 | 132871 | 303350 | 303283 | / |
| mtpt38 | 68 | 98.529 | 1 | 0 | 85790 | 85857 | 303283 | 303350 | / |
| mtpt39 | 76 | 93.421 | 4 | 1 | 52425 | 52499 | 17563 | 17488 | *trnC-CAU* |
| mtpt40 | 97 | 82.474 | 17 | 0 | 121313 | 121409 | 115532 | 115436 | / |
| mtpt41 | 97 | 82.474 | 17 | 0 | 97252 | 97348 | 115436 | 115532 | / |
| mtpt42 | 97 | 82.474 | 17 | 0 | 97252 | 97348 | 388682 | 388586 | / |
| mtpt43 | 97 | 82.474 | 17 | 0 | 121313 | 121409 | 388586 | 388682 | / |
| mtpt44 | 80 | 86.25 | 8 | 3 | 105121 | 105200 | 332599 | 332675 | / |
| mtpt45 | 43 | 90.698 | 4 | 0 | 7504 | 7546 | 59527 | 59485 | / |
| mtpt46 | 35 | 97.143 | 0 | 1 | 123265 | 123298 | 235277 | 235243 | / |
| mtpt47 | 35 | 97.143 | 0 | 1 | 95363 | 95396 | 235243 | 235277 | / |
| mtpt48 | 30 | 100 | 0 | 0 | 87906 | 87935 | 305394 | 305365 | / |
| mtpt49 | 30 | 100 | 0 | 0 | 130726 | 130755 | 305365 | 305394 | / |

**Supplementary Table 8.** The abbreviations and NCBI accession numbers of mitogenomes used in this study.

| **Species** | **Family** | | **Accession Numbers** |
| --- | --- | --- | --- |
| *Zea luxurians* | | Poaceae | NC_008333.1 |
| *Vitis vinifera* | | Vitaceae | NC_012119.1 |
| *Oryza rufipogon* | | Poaceae | NC_013816.1 |
| *Phoenix dactylifera* | | Arecaceae | NC_016740.1 |
| *Spirodela polyrhiza* | | Araceae | NC_017840.1 |
| *Oryza minuta* | | Poaceae | NC_029816.1 |
| *Cocos nucifera* | | Arecaceae | NC_031696.1 |
| *Arabidopsis thaliana* | | Brassicaceae | NC_037304.1 |
| *Eleusine indica* | | Poaceae | NC_040989.1 |
| *Chrysopogon zizanioides* | | Poaceae | NC_056367.1 |
| *Thinopyrum obtusiflorum* | | Poaceae | OK120846.1 |
| *Aegilops speltoides var. ligustica* | | Poaceae | AP013107.1 |
| *Bambusa oldhamii* | | Poaceae | EU365401.1 |
| *Coix lacryma-jobi var. puellarum* | | Poaceae | MT471098.1 |
| *Ferrocalamus rimosivaginus* | | Poaceae | JN120789.1 |
| *Ginkgo biloba* | | Ginkgoaceae | KM672373.1 |
| *Hordeum vulgare subsp. vulgare* | | Poaceae | MN127966.1 |
| *Lolium perenne* | | Poaceae | JX999996.1 |
| *Oryza coarctata* | | Poaceae | MG429050.1 |
| *Oryza sativa Indica Group* | | Poaceae | JF281153.1 |
| *Sorghum bicolor* | | Poaceae | NC_008360.1 |
| *Sporobolus alterniflorus* | | Poaceae | MT471321.1 |
| *Tripsacum dactyloides* | | Poaceae | NC_008362.1 |
| *Triticum aestivum* | | Poaceae | NC_036024.1 |
| *Zea perennis* | | Poaceae | NC_008331.1 |
| *Gastrodia elata* | | Orchidaceae | MF070086.1—MF070085.1 |
| *Hemerocallis citrina* | | Asphodelaceae | MZ726801.1—MZ726803.1 |
| *Amborella trichopoda* | | Amborellaceae | KF754802.1—KF754803.1 |
| *Avena longiglumis* | | Poaceae | OP649434.1—OP649437.1 |

**Supplementary Figure 1.** Protein-coding genes annotated in *Z. latifolia* mitochondrial genome in comparison to related species.

**
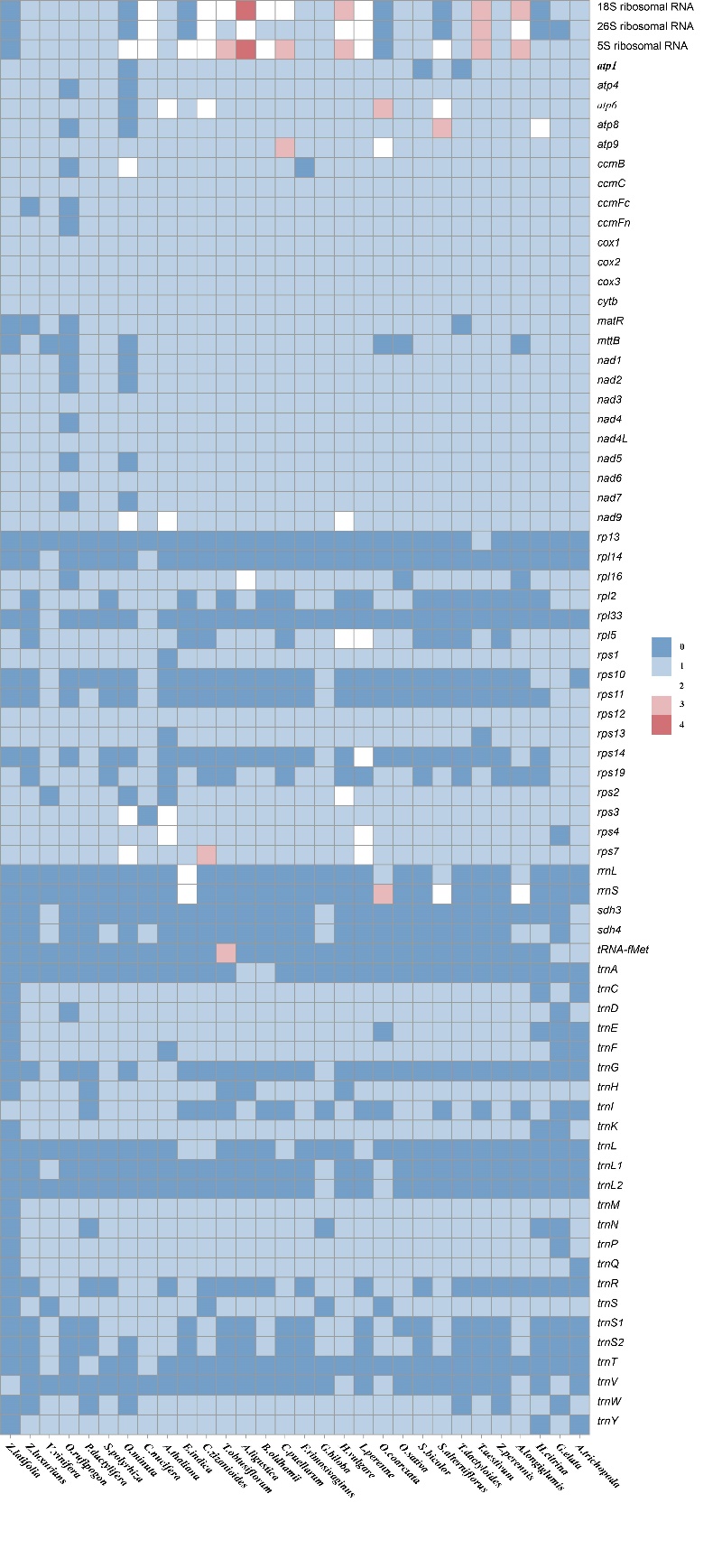
**
